# ROIV-SLAM: Rotation-Optimized Inertial–Visual SLAM for a Non-Coaxial Two-Wheeled Robot Under Roll Disturbances

**DOI:** 10.3390/s26134053

**Published:** 2026-06-25

**Authors:** Chong Feng, Cheng Ren, Wenbo Gao, Zhan Shi, Chunjuan Bo, Chang Kou, Zhun Feng

**Affiliations:** 1 College of Information and Communication, Dalian Minzu University, Dalian 116600, China; r17806295348@163.com (C.R.); g123029g@gmail.com (W.G.); sz1134@163.com (Z.S.); bcj@dlnu.edu.cn (C.B.); 2 Dalian Everyday Good Electronic Co., Ltd., Tieshan Dongsan Road, Dalian 116630, China; stonebrother@dlrijia.com; 3 Industry–Education Integration Center, Liaoning Railway Vocational and Technical College, Jinzhou 121000, China; fengzhun@lntdxy.edu.cn

**Keywords:** rotation optimization, visual–inertial fusion, SLAM, motion disturbances, non-coaxial two-wheeled robot

## Abstract

To address the problem of high-frequency roll disturbances generated during dynamic balancing in non-coaxial two-wheeled robots, this paper proposes a Rotation-Optimized Inertial–Visual SLAM system (ROIV-SLAM) for robust state estimation. The proposed approach adopts a decoupled architecture for translation and rotation estimation. In the front-end, an Extended Kalman Filter (EKF) is employed to fuse LiDAR, an inertial measurement unit (IMU), and wheel odometry to obtain an initial translation estimate. Meanwhile, a physical manifold constraint is constructed using the gravity vector and surface normals extracted from RGB-D point clouds, supporting stable rotation estimation under high-frequency disturbances through Lie-group-based optimization. In the back-end, a factor graph is established, and loop closure robustness is enhanced through vision–LiDAR scan matching. Experimental results indicate that ROIV-SLAM achieves improved trajectory consistency with respect to the optimized reference trajectory and more robust mapping performance compared with the evaluated baseline approaches in the tested scenarios. The results further suggest that introducing task-specific physical dynamic constraints and a decoupled estimation mechanism helps suppress high-frequency motion noise inherent to balancing robots, thereby improving the robustness of state estimation in complex environments.

## 1. Introduction

With the advancement of mobile robotics from controlled laboratory environments to complex outdoor scenarios, Simultaneous Localization and Mapping (SLAM) has become a fundamental technology for enabling autonomous navigation. However, the robustness and accuracy of SLAM systems face increasingly stringent challenges in such environments. Conventional wheeled robots are typically designed under the assumption of operating on relatively flat terrain. In contrast, platforms with special dynamic characteristics—such as non-coaxial two-wheeled robots and humanoid wheel–leg robots—experience continuous high-frequency attitude adjustments and significant body oscillations during motion. In unstructured or rough terrains in particular, overcoming sensor observation noise caused by severe body motion while maintaining long-term and high-precision state estimation has become a critical research problem in robotics.

Unlike conventional four-wheeled vehicles or standard wheeled platforms, non-coaxial two-wheeled robots exhibit an inverted pendulum structure and must continuously adjust their roll angle at high frequency to maintain dynamic balance. Such self-balancing motion introduces a form of high-frequency disturbance to SLAM systems. This disturbance differs from passive attitude perturbations caused by terrain irregularities and from the intermittent posture changes observed in legged robots during gait transitions. High-frequency roll disturbances simultaneously degrade the observations of multiple sensors:

1. LiDAR sensors rely on sequential scanning mechanisms. If rapid body rotation occurs during a single point cloud acquisition cycle, the resulting point cloud becomes distorted. Traditional de-distortion methods typically rely on high-rate IMU interpolation or constant-velocity assumptions. However, under severe oscillations of two-wheeled robots, IMU measurement noise is amplified and the constant-velocity assumption becomes invalid, leading to layered artifacts or ghosting during point cloud registration.
2. IMU sensors suffer from bias and random walk effects. Under intense vibration conditions, integration errors accumulate rapidly, producing significant attitude drift within a short time period.
3. Cameras are susceptible to motion blur during rapid rotation, resulting in reduced feature descriptor consistency and increased failure rates in optical flow tracking.

Beyond corrupted observations, error leakage caused by strong coupling between rotation and translation represents the most fundamental mathematical challenge in highly dynamic scenarios. In standard 6-DoF SLAM optimization, the system state includes both rotational and translational components, and the objective function is typically formulated as a nonlinear least-squares problem.

(1) $$R_{t}^{*}=\arg\min_{R_{t}}\sum_{i}{∥f\left(z_{i},R_{t}\right)∥}^{2}$$

Under small motions, this optimization problem is usually well-conditioned. However, in highly dynamic scenarios involving two-wheeled robots—especially when distant feature points are observed—even a small rotational error can be amplified into a large translational residual due to the long lever arm effect. As a result, the optimizer struggles to distinguish the true source of the residual and may incorrectly adjust the translation component to compensate for rotational errors. For example, pure in-place rotation may be misinterpreted as lateral translation, leading to severe trajectory distortion. Since the joint optimization space is non-convex and the initial estimate may deviate from the true state, iterative optimization is more likely to converge to local minima.

It should be noted that the objective of this work differs from that of modern 3D LiDAR–inertial or LiDAR–visual–inertial SLAM systems such as LIO-SAM, FAST-LIO2, LVI-SAM, and R3LIVE [1,2,3]. These frameworks are designed for platforms equipped with 3D LiDAR sensors and generally rely on dense three-dimensional geometric observations. More recently, robust multi-sensor fusion systems have increasingly focused on improving state estimation under aggressive motion, degraded observations, and platform-specific motion constraints. FAST-LIVO2 extends tightly coupled LiDAR–visual–inertial fusion by integrating heterogeneous observations within an efficient iterated filtering framework [4]. In addition, wheel-assisted visual–inertial odometry systems have demonstrated that introducing wheel, ground-contact, and vehicle-motion constraints can significantly improve observability and reduce drift accumulation under restricted motions and dynamic disturbances [5,6]. These developments highlight the importance of incorporating physically meaningful motion priors into state estimation, particularly for robotic platforms whose body dynamics strongly affect sensor observations. In contrast, the target platform considered in this work is a low-cost non-coaxial self-balancing robot equipped only with a 2D planar LiDAR and an RGB-D camera. Moreover, the proposed framework differs fundamentally from ROLO-SLAM [7]. ROLO-SLAM focuses on rotation–translation decoupling for ground vehicles operating on uneven terrain using LiDAR observations. In contrast, ROIV-SLAM addresses a different sensing and dynamic setting, namely a non-coaxial self-balancing two-wheeled robot subject to continuous high-frequency roll disturbances. The proposed method introduces gravity- and ground-normal-based attitude constraints together with a physically bounded roll angle smoothing factor, which are specifically designed to improve rotational observability and robustness under balancing-induced disturbances. Therefore, the proposed method focuses on improving localization robustness under severe roll disturbances within the constraints of a lightweight 2D LiDAR–vision sensing configuration.

To address the aforementioned challenges, this paper proposes ROIV-SLAM (Rotation-Optimized Inertial–Visual SLAM), a lightweight multi-sensor fusion framework specifically designed for non-coaxial self-balancing robots operating under severe roll disturbances. Instead of treating the roll angle as a conventional state variable to be estimated freely, we model it as a core state strongly constrained by the gravity vector and the ground normal vector. A translation–rotation decoupled front-end architecture is designed to prioritize accurate rotation estimation on the manifold space. Meanwhile, the back-end factor graph introduces a roll angle smoothing factor and a vision–LiDAR dual-validation loop closure constraint, improving localization robustness and trajectory consistency under high-frequency roll disturbances.

The main contributions of this work are summarized as follows:

1. A rotation-aware multi-sensor fusion SLAM framework (ROIV-SLAM) is proposed for non-coaxial two-wheeled robots operating under continuous high-frequency roll disturbances.
2. A decoupled front-end state estimation architecture is developed, where translational motion is estimated through EKF-based multi-sensor fusion, while rotational motion is optimized on the SO(3) manifold using physically meaningful attitude constraints.
3. A joint attitude constraint model that combines gravity vectors and RGB-D ground normals is introduced to improve rotational observability and attitude stability under severe oscillatory motion.
4. A roll angle smoothing factor is incorporated into the sliding-window factor graph, together with a vision–LiDAR dual-validation loop closure mechanism, improving trajectory consistency, loop-closure reliability, and global mapping robustness.

## 2. Materials and Methods

### 2.1. Development of LiDAR and Visual SLAM

Early SLAM research primarily focused on single-sensor modalities. In LiDAR-based SLAM, the LOAM framework proposed by Zhang et al. established a feature-based odometry architecture by extracting planar and edge features for efficient pose estimation. Subsequent works further improved this framework [8]. For example, LeGO-LOAM introduced ground segmentation to enhance adaptability in complex environments [9], while SuMa++ incorporated semantic information to remove dynamic objects and improve the static consistency of generated maps [10]. However, pure LiDAR-based approaches tend to suffer from drift in geometrically degenerate environments such as long corridors or open areas, and they are particularly sensitive to motion distortion caused by rapid rotational movements.

In the field of visual SLAM, systems such as ORB-SLAM and VINS-Mono represent typical frameworks based on sparse feature methods. These approaches can achieve high localization accuracy in environments with rich visual textures [11,12]. Nevertheless, visual sensors are highly sensitive to illumination changes and are prone to motion blur during rapid motion, which reduces feature consistency and tracking reliability. As a result, purely vision-based systems are often insufficient to support robust all-weather and high-dynamic robotic operations.

### 2.2. Evolution of Multi-Sensor Fusion Techniques

The limitations of single-sensor systems have driven the adoption of multi-sensor fusion as a mainstream paradigm in SLAM research. Current approaches can be broadly categorized into tightly coupled LiDAR–inertial systems, visual fusion systems, lightweight multi-modal frameworks, and robust back-end optimization techniques.

Representative frameworks such as LIO-SAM and FAST-LIO2 leverage high-frequency IMU measurements through preintegration to handle rapid motion and short-term sensor degradation [1,2]. FAST-LIO2 further enhances odometry efficiency using an error-state Kalman filter (ESKF) combined with the ikd-Tree data structure, increasing system bandwidth [2,13]. Recent works like OR-LIM explicitly incorporate observability and motion stability constraints to improve robustness under high dynamic motions, jointly optimizing sensor poses and scene geometry even in perceptually limited environments [14].

Visual–inertial frameworks augmented with LiDAR, such as LVI-SAM and R3LIVE, exploit the complementary strengths of LiDAR geometric accuracy, visual texture information, and IMU dynamic sensing. These systems achieve improved localization and mapping performance by integrating multiple sensing modalities in a tightly coupled manner [3,15].

For resource-constrained platforms, lightweight fusion architectures have been explored. FAST-LIVO2 combines LiDAR, visual, and inertial measurements while maintaining real-time performance in large-scale environments [4]. Similarly, wheel-assisted and ground-constrained visual–inertial systems demonstrate that incorporating physically meaningful motion constraints can enhance localization robustness when geometric observability is limited [5,6]. However, reliance on dense 3D LiDAR limits applicability to platforms equipped only with 2D LiDAR sensors.

Even well-designed multi-sensor fusion frameworks require robust back-end optimization when observations are contaminated by outliers. Techniques such as switchable constraints or maximum mixture models, exemplified by the works of Latif et al. and TEASER, effectively remove incorrect loop closures and ensure correct global map topology [16,17].

The sensing configuration considered in this study presents a unique challenge: achieving robust SLAM under restricted geometric observability and severe roll oscillations. Unlike typical handheld or vehicular platforms, our focus is on scenarios where conventional dense 3D LiDAR observations are unavailable, requiring careful design of both front-end fusion and back-end optimization.

### 2.3. SLAM Optimization for Special Kinematics and Terrains

Conventional SLAM frameworks typically assume smooth robot motion or model the robot as a rigid body with generic 6-DoF estimation. However, such assumptions are not always valid for platforms with special kinematic structures. On rough terrain, large roll and pitch variations of the robot body can cause severe nonlinear distortions in point clouds, making direct joint optimization prone to vertical drift or convergence to local minima. ROLO-SLAM introduced a front-end architecture based on rotation–translation decoupling, where rotational degrees of freedom are first estimated independently before solving for translation. This strategy effectively alleviates localization difficulties caused by terrain undulations [7].

Similar ideas have also been explored in wheel-assisted and ground-constrained state estimation. VINS on Wheels demonstrated that wheel encoder measurements can improve scale observability and estimation consistency for ground robots under restricted motion conditions [5]. Ground-VIO further exploited camera–ground geometric constraints to enhance monocular visual–inertial odometry by introducing online estimation of ground-related parameters [6]. More recently, RGB-D–wheel SLAM systems have introduced planar motion constraints into graph optimization for two-wheeled mobile robots, demonstrating that wheel- and motion-prior constraints can improve robustness and map consistency in indoor environments [18]. In addition, recent visual–inertial–wheel odometry studies have emphasized the importance of wheel-slip handling, online calibration, and observability analysis for reliable localization on wheeled platforms [19,20]. These studies suggest that explicitly incorporating platform-specific physical constraints into SLAM optimization can substantially improve estimation robustness.

For wheeled–legged robots or platforms with explicit contact constraints, incorporating the robot’s kinematic model into SLAM can further improve estimation accuracy. Previous studies have shown that contacts between the robot’s feet or wheels and the ground naturally form manifold constraints. These constraints can be formulated as ground-normal residuals and integrated into factor graph optimization. Combined with kinematic odometry, such physical constraints help suppress vertical drift and improve pose estimation stability, demonstrating the importance of task-specific physical priors for robots operating under special kinematics.

### 2.4. Research Gap and Motivation

Although recent studies have investigated wheel-assisted visual–inertial odometry, ground-constrained VIO, RGB-D–wheel SLAM, and LiDAR–visual–inertial fusion for mobile robots [4,5,6,18,19], robust SLAM systems specifically designed for dynamically unstable self-balancing platforms remain relatively limited. Existing methods primarily target conventional wheeled vehicles or general mobile robots and do not explicitly address continuous balancing-induced roll oscillations.

1. **Unique characteristics of high-frequency attitude disturbances.** The self-balancing mechanism requires continuous high-frequency roll adjustments to maintain stability, introducing disturbances that are active, continuous, and high-frequency in nature.
2. **Lack of absolute rotation constraints.** In conventional LIO/VIO systems, gyroscope integration errors accumulate rapidly under severe oscillations. Meanwhile, existing ground constraints do not fully exploit visual or depth-based surface normals for direct attitude correction.
3. **Insufficient utilization of motion smoothness priors.** Although the balancing process involves rapid motion, it still follows smooth and continuous dynamic behavior. Existing back-end optimization frameworks mainly focus on removing discrete false loop closures, while explicit constraints enforcing the temporal smoothness of robot motion are rarely considered.

To address these issues, this paper proposes **ROIV-SLAM**. The roll angle is treated as a core state variable under strong physical constraints derived from gravity vectors and ground normals. By prioritizing rotation estimation on the manifold space and introducing a roll angle smoothing factor in the back-end factor graph, the proposed framework improves localization robustness under severe roll disturbances caused by high-dynamic balancing motions.

## 3. System Overview

ROIV-SLAM consists of four major components: multi-sensor data preprocessing and spatiotemporal calibration, front-end decoupled estimation, back-end factor graph optimization, and loop closure detection.

First, raw measurements from LiDAR, RGB-D cameras, IMU, and wheel odometry are preprocessed, and both spatial extrinsic parameters and temporal synchronization offsets among sensors are calibrated.

The front-end adopts a translation–rotation decoupled estimation strategy. Translational motion is estimated using an Extended Kalman Filter (EKF), where IMU and wheel odometry provide motion prediction, while LiDAR scan matching serves as the external observation for correction. In contrast, rotational motion is optimized on the SO(3) manifold using physical constraints derived from the gravity vector and the ground normal extracted from RGB-D observations. This Lie-group-based optimization enables accurate attitude estimation under high-frequency roll disturbances.

Afterward, translation and rotation estimates are synchronized using timestamps and composed into a full 6-DoF pose together with its covariance, which is then used as the initial estimate for the back-end optimization.

The back-end employs a sliding-window factor graph for joint optimization. Multiple factors are incorporated, including IMU preintegration factors, wheel odometry factors, visual matching factors, scan-to-submap factors, loop closure constraints, and a roll angle smoothness factor that enforces temporal consistency of the balancing motion.

**Figure 1:**
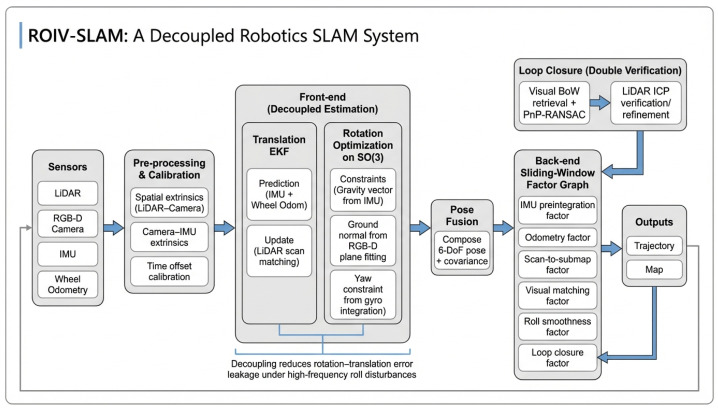
Overview of the proposed ROIV-SLAM framework. The system consists of sensor preprocessing and calibration, a decoupled front-end for translation and rotation estimation, pose fusion, and a back-end sliding-window factor graph with dual-validation loop closure detection.

For loop closure detection, a dual-validation mechanism is adopted. Candidate loops are first retrieved using visual bag-of-words matching and geometrically verified via PnP-RANSAC. Subsequently, LiDAR-based scan matching performs a secondary verification and refinement, improving the robustness of loop detection and ensuring global map consistency. The overall architecture of the proposed ROIV-SLAM framework is shown in Figure 1.

## 4. Methods

### 4.1. Kinematic Model of the Non-Coaxial Two-Wheeled Robot

To describe the motion characteristics of the non-coaxial two-wheeled robot, a kinematic model and corresponding coordinate systems are established.

**Global Coordinate Frame.** The global coordinate frame is defined as a fixed world reference frame used to represent the absolute pose of the robot. The robot pose in this frame is expressed by the planar position (X,Y) and the heading angle θ.

**Body Coordinate Frame.** The body coordinate frame is rigidly attached to the robot. The origin of this frame is located at the midpoint between the front and rear wheel centers. The *x*-axis points along the forward motion direction of the robot, while the *y*-axis is perpendicular to the forward direction within the ground plane.

**Figure 2:**
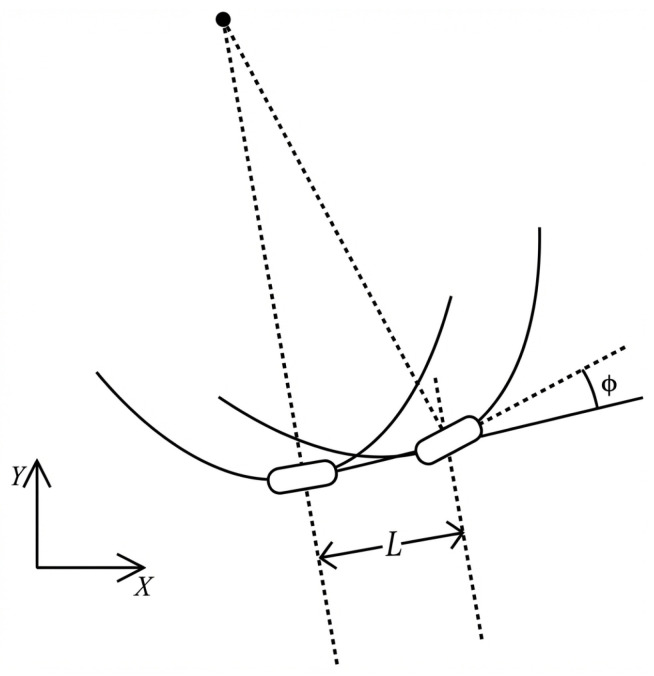
Kinematic model of the self-balancing two-wheeled robot. The figure illustrates the coordinate frames, state variables, and motion relationships used for system modeling and state estimation.

Unlike conventional differential-drive robots, non-coaxial two-wheeled robots must continuously regulate their roll angle to maintain dynamic balance during motion, resulting in inherently three-dimensional and highly nonlinear behavior. Therefore, the two-dimensional planar kinematic model established in this study is not intended to fully capture the system’s dynamic behavior, but rather to serve as a low-dimensional approximation for characterizing the robot’s short-term translational trends. The coordinate definitions and kinematic relationship of the robot are illustrated in Figure 2.

It should be clarified that the non-coaxial self-balancing two-wheeled robot used in this work is equipped with a front-wheel steering mechanism. Therefore, its dominant planar motion is consistent with the kinematic structure of a front-wheel-steered vehicle.

The momentum wheel is introduced for dynamic balance control and primarily affects the roll stabilization of the robot body. It does not alter the geometric relationship between forward velocity, steering angle, yaw rate, and planar pose evolution that characterizes front-wheel steering motion. Consequently, the bicycle model is adopted to describe the planar kinematic constraints of the robot during short-term state propagation.

In this work, the bicycle model is not intended to represent the complete balancing dynamics of the robot. Instead, it serves as a low-dimensional motion prior for the EKF prediction stage. Roll disturbances and attitude fluctuations are handled separately through IMU measurements, SO(3)-based rotation optimization, gravity- and ground-normal-based attitude constraints, and the roll angle smoothing factor introduced in the backend optimization.

Accordingly, the planar motion of the robot is approximated using a bicycle-model formulation. Let the robot pose be (x,y,θ), where (x,y) represents the planar position and θ denotes the heading angle. The angular velocity of the rear wheel is denoted by $w_{r}$, the steering angle by δ, the wheelbase (distance between the front and rear wheels) by *L*, and the wheel radius by *r*.

The linear velocity *v* of the robot body is generated by the rotation of the rear wheel. Under the no-slip assumption, the forward velocity of the rear wheel center equals the product of its angular velocity and the wheel radius. Therefore, the linear velocity can be expressed as

(2) $$v=rw_{r}$$

The angular velocity ω represents the rate of change of the heading angle θ, which is determined jointly by the steering angle δ and the linear velocity *v*.

Considering the right triangle formed by the rear wheel center, the front wheel center, and the instantaneous center of rotation, the turning radius *R* satisfies the geome- tric relationship

(3) $$\tan\delta =\frac{L}{R}$$

Since the turning radius can also be expressed using the linear and angular velocities

(4) $$R=\frac{v}{\omega }$$

substituting this expression into the geometric relationship yields

(5) $$\tan\delta =\frac{L}{v/\omega }=\frac{L\omega }{v}$$

Solving for the angular velocity gives

(6) $$\omega =\frac{v}{L}\tan\delta$$

Substituting $v=rw_{r}$ leads to

(7) $$\omega =\frac{r\;w_{r}}{L}\tan\delta$$

Combining the expressions of linear velocity and angular velocity, the pose dynamics of the robot in the global coordinate frame can be written as

(8) $$\left[\begin{array}{c} \dot{x}\\ \dot{y}\\ \dot{\theta } \end{array}\right]=\left[\begin{array}{c} v\cos\theta \\ v\sin\theta \\ \omega \end{array}\right]$$

Substituting the expressions of *v* and ω yields the final kinematic model of the robot

(9) $$\left[\begin{array}{c} \dot{x}\\ \dot{y}\\ \dot{\theta } \end{array}\right]=\left[\begin{array}{c} (rw_{r})\cos\theta \\ (rw_{r})\sin\theta \\ \frac{rw_{r}}{L}\tan\delta \end{array}\right]$$

It should be noted that, due to the continuous roll oscillations exhibited by non-coaxial two-wheeled robots during operation, the planar motion model inevitably introduces truncation errors and model mismatch. In particular, under conditions of high-frequency attitude variations, wheel speed measurement errors and attitude coupling effects can be significantly amplified, thereby degrading pose estimation accuracy.

Accordingly, this model is employed only as prior information for short-term translational prediction and is not intended to describe the complete balancing dynamics of the robot. Its primary role is to provide a low-dimensional motion prior for the EKF prediction stage, transforming wheel speed measurements into a continuous translational estimate and enabling covariance propagation under the assumption of locally smooth planar motion.

The effects of roll oscillations, attitude disturbances, and balance-control dynamics are explicitly handled through IMU measurements, the SO(3)-based rotation optimization framework, the gravity- and ground-normal-based attitude constraints, and the roll angle smoothing factor introduced in the backend optimization. Therefore, the bicycle model serves only as a planar kinematic approximation and is not responsible for modeling the full three-dimensional balancing behavior of the robot.

Furthermore, the limitations of the bicycle model also highlight the inadequacy of standalone odometry for this application, thereby motivating the need for the proposed multi-sensor fusion framework and the translation–rotation decoupling strategy developed in the subsequent sections.

### 4.2. Translation Estimation Based on EKF

Under high-frequency roll disturbances, if rotation and translation are still estimated in a strongly coupled manner, rotational errors can propagate into the translational states through the observation residuals, leading to non-physical distortions in the estimated trajectory. To mitigate such error leakage, this study models translation estimation as an independent low-dimensional recursive filtering problem. Short-term predictions are provided by the IMU and wheel odometry, while LiDAR-based geometric observations are used for state correction. This approach yields continuous, smooth translational estimates with explicit uncertainty representation.

Recent multi-sensor SLAM systems have shown that tightly coupled visual, inertial, and LiDAR measurements can significantly improve robustness in challenging environments. For example, TC2LI-SLAM integrates camera, LiDAR, and IMU measurements with a separated front-end and a unified back-end optimization framework [21]. Similarly, recent LiDAR-visual-inertial systems formulate LiDAR, visual, and inertial constraints in a factor graph to improve pose accuracy and large-scale consistency [22]. For ground vehicles, motion-prior-based formulations such as SE(2)-constrained LiDAR-inertial odometry have further demonstrated that incorporating platform-specific physical constraints can improve localization robustness [23]. High-frequency IMU measurements and short-term drift-free wheel odometry are used for state prediction, providing a continuous and smooth motion estimate. Meanwhile, LiDAR scan matching is insensitive to illumination changes and provides reliable geometric constraints. Therefore, the accurate external observation obtained from 2D LiDAR is used to correct the accumulated error in the prediction stage.

#### 4.2.1. State Vector Definition

To explicitly characterize the uncertainty of the prediction model, the process noise is assumed to follow a zero-mean Gaussian distribution. The process noise mainly originates from IMU measurement noise, wheel-odometry uncertainty, and IMU bias random walks.

The process noise vector is defined as

(10) $$w_{k}={\left[\begin{array}{ccccccc} n_{ax} & n_{ay} & n_{\omega z} & n_{odo} & n_{bax} & n_{bay} & n_{bwz} \end{array}\right]}^{T}$$

where $n_{ax}$ and $n_{ay}$ denote the accelerometer noise components, $n_{\omega z}$ denotes the gyroscope noise, $n_{odo}$ represents wheel-odometry uncertainty, and $n_{bax}$, $n_{bay}$, and $n_{bwz}$ correspond to the random-walk noise of the IMU biases.

Accordingly, the process noise covariance matrix is modeled as

(11) $$Q_{k}=\mathrm{diag}\left(\sigma_{ax}^{2},\sigma_{ay}^{2},\sigma_{\omega z}^{2},\sigma_{odo}^{2},\sigma_{bax}^{2},\sigma_{bay}^{2},\sigma_{bwz}^{2}\right)$$

The LiDAR scan-matching observation noise is also assumed to follow a zero-mean Gaussian distribution

(12) $$v_{k}∼N\left(0,R_{k}\right)$$

where $R_{k}$ denotes the observation noise covariance matrix determined by the scan-matc- hing quality.

The system state to be estimated consists of the robot pose and IMU biases, defined as

(13) $$x={\left[\begin{array}{cccccc} p_{x} & p_{y} & \theta & b_{ax} & b_{ay} & b_{wz} \end{array}\right]}^{T}$$

where $p_{x}$ and $p_{y}$ represent the robot position in the world frame, θ denotes the robot heading angle, $b_{ax}$ and $b_{ay}$ represent the IMU accelerometer biases along the *x* and *y* axes, and $b_{wz}$ denotes the gyroscope bias along the *z* axis.

#### 4.2.2. Prediction Stage

The prediction stage utilizes high-frequency IMU measurements and wheel odometry data to propagate the robot state forward in time and update its uncertainty.

IMU provides acceleration and angular velocity measurements, which can be integrated to estimate velocity and position changes. However, IMU measurements suffer from bias and noise, leading to drift over time. Wheel odometry provides relative displacement information with lower frequency but higher short-term stability. By combining IMU and odometry data, the high-frequency IMU measurements fill the temporal gaps between odometry readings, while odometry helps correct the short-term drift of IMU.

The motion model describes how the state evolves over time.

The predicted state is given by

(14) $${\hat{x}}_{k|k-1}={\hat{x}}_{k-1|k-1}+f\left({\hat{x}}_{k-1|k-1},u_{k-1}\right)\Delta t$$

where $u_{k-1}$ represents the input consisting of IMU and odometry measurements.

The predicted covariance is

(15) $$P_{k|k-1}=F_{k}P_{k-1|k-1}F_{k}^{T}+Q_{k}$$

where $F_{k}$ is the Jacobian of the state transition model, describing how the state evolves linearly over time, and $Q_{k}$ is the process noise covariance matrix determined mainly by IMU noise and bias random walk. The state prediction equation recursively estimates the prior pose at the current time step using IMU measurements and wheel odometry, while the covariance propagation equation characterizes the uncertainty of this prediction, providing the basis for Kalman gain computation in the subsequent measurement update.

The translational prediction is primarily determined by the IMU-measured acceleration and the displacement inferred from odometry. Specifically, IMU acceleration is integrated to obtain velocity and position, whereas odometry directly provides velocity and position estimates. These two sources are fused according to their respective reliability weights to produce a more robust prediction. However, due to inherent drift in the IMU, the predicted state gradually deviates from the reference trajectory over time, necessitating correction through measurement updates.

#### 4.2.3. Update Stage

When LiDAR data becomes available, the EKF enters the update stage. Instead of directly providing absolute position measurements, LiDAR produces a set of precise environmental observations. By matching the current LiDAR scan with the previous scan, a relative pose transformation is obtained. This relative transformation can be converted into an absolute pose observation in the world frame.

It should be clarified that the experimental platform used in this work is equipped with a 2D LiDAR sensor. Therefore, the scan matching process is performed using planar laser scans rather than full 3D point clouds.

The 2D LiDAR scan matching mainly provides geometric constraints on planar translation and yaw rotation. Specifically, scan matching is carried out in the horizontal plane to estimate the relative planar transformation between consecutive scans or between the current scan and the local map.

Consequently, the LiDAR observation used in the EKF update is defined only in terms of planar position and heading, namely $\left(p_{x},p_{y},\theta \right)$. Since a 2D LiDAR cannot directly observe three-dimensional geometric structures, it does not provide independent constraints on roll and pitch motion.

Therefore, roll and pitch estimation are handled separately by the rotation estimation module described in the following section, where IMU measurements, RGB-D ground-normal observations, and Lie-group-based optimization are jointly utilized to estimate the full attitude of the robot.

The observation model is

(16) $$z_{k}=h\left(x_{k}\right)+v_{k}$$

where $z_{k}$ represents the LiDAR observation

(17) $$z_{k}={\left[\begin{array}{ccc} p_{x}^{laser} & p_{y}^{laser} & \theta^{laser} \end{array}\right]}^{T}$$

and $h(x_{k})$ maps the state vector to the observation space

(18) $$h\left(x_{k}\right)={\left[\begin{array}{ccc} p_{x} & p_{y} & \theta \end{array}\right]}^{T}$$

while $v_{k}$ denotes the observation noise.

The Kalman gain is computed as

(19) $$K_{k}=P_{k|k-1}H_{k}^{T}{\left(H_{k}P_{k|k-1}H_{k}^{T}+R_{k}\right)}^{-1}$$

where $H_{k}$ is the Jacobian of the observation model and $R_{k}$ represents the LiDAR observation noise covariance.

The state update is

(20) $${\hat{x}}_{k|k}={\hat{x}}_{k|k-1}+K_{k}\left(z_{k}-h\left({\hat{x}}_{k|k-1}\right)\right)$$

where $z_{k}-h\left({\hat{x}}_{k|k-1}\right)$ is the measurement residual.

The covariance update is

(21) $$P_{k|k}=\left(I-K_{k}H_{k}\right)P_{k|k-1}$$

If the IMU and odometry prediction interval becomes longer, $P_{k|k-1}$ increases, indicating higher uncertainty. Consequently, the Kalman gain $K_{k}$ increases, and the EKF relies more heavily on LiDAR observations to correct accumulated drift. Similarly, if the LiDAR observation noise $R_{k}$ is small, the Kalman gain also increases, allowing LiDAR measurements to have a stronger influence on the state estimate.

During the measurement update stage, LiDAR-based geometric constraints are employed to correct the drift from inertial integration and errors in wheel odometry. The observation noise covariance is adaptively adjusted according to the quality of point cloud matching: in feature-rich environments, the weight of LiDAR observations is increased, whereas in degenerate scenarios, the prediction model is given greater influence. This adaptive strategy enhances the overall robustness of the state estimation.

Through this process, EKF continuously corrects the drift accumulated from IMU and odometry predictions using precise LiDAR observations, achieving accurate, robust, and real-time translation estimation.

### 4.3. Rotation Estimation Based on Lie-Group Optimization

Recent visual SLAM reviews have also emphasized that rapid camera motion, weak texture, illumination variation, and dynamic scene changes remain major causes of tracking degradation, which motivates the introduction of additional inertial and geometric constraints in visually challenging conditions [24]. Traditional approaches based on frame-to-frame matching or IMU integration often fail or produce large drift when the roll angle changes drastically. In this work, the rotation estimation module exploits two complementary observations of the vertical direction: the gravity vector measured by the IMU and the ground normal estimated from RGB-D point clouds. Although these two vectors are geometrically related in ideal flat-ground conditions, they are obtained from different sensing modalities and therefore provide complementary evidence for stabilizing roll and pitch estimation under high-frequency disturbances. These vectors are used to constrain and estimate the rotation matrix between the robot body frame and the horizontal coordinate frame. It should be emphasized that, in non-coaxial two-wheeled robot systems, the accuracy of rotation estimation directly determines the reliability of translation estimation. When rotational errors are present, the LiDAR point cloud undistortion and coordinate transformation processes introduce systematic biases, which are further amplified into spurious translational errors through geometric constraints. Therefore, it is necessary to prioritize high-precision rotation estimation and model it independently from the front-end estimation process. The IMU directly measures the gravity vector in the body frame. For the RGB-D camera, the ground normal vector is estimated by first filtering out depth points that are too far or too close, and then applying RANSAC plane fitting to obtain the ground plane normal vector in the robot body frame.

In the ideal horizontal world frame, the gravity vector direction is fixed as

$$g_{world}={\left[0,0,-1\right]}^{T}$$

and the ground normal direction is

$$n_{world}={\left[0,0,1\right]}^{T}.$$

The optimal rotation matrix *R* is solved such that the measured gravity vector $g_{imu}$ and ground normal vector $n_{rgbd}$ in the body frame are rotated into the horizontal coordinate frame and become as close as possible to their ideal values.

Let the rotation matrix R∈SO(3) transform a vector $v_{b}$ in the body frame to the world frame

(22) $$v_{w}=Rv_{b}$$

The rotation matrix can be decomposed into rotations around the *X* (roll), *Y* (pitch), and *Z* (yaw) axes

(23) $$R=R_{z}\left(\psi \right)R_{y}\left(\phi \right)R_{x}\left(\alpha \right)$$

where α is the roll angle, ϕ is the pitch angle, and ψ is the yaw angle.

From an observability perspective, the gravity vector and the RGB-D ground normal mainly constrain roll and pitch, whereas yaw rotation around the vertical direction remains unobservable from these measurements alone. Therefore, gyroscope integration is introduced as a short-term relative yaw prior, while long-term yaw consistency is maintained by LiDAR scan matching, visual constraints, loop closure, and back-end graph optimization.

The objective function is defined as the weighted sum of three residual terms

(24) $$\arg\min_{\delta \theta }L\left(\delta \theta \right)=W_{g}∥e_{g}{∥}^{2}+W_{n}∥e_{n}{∥}^{2}+W_{\psi }{∥e_{\psi }∥}^{2}$$

where the rotation update is represented using the Lie algebra increment

$$\delta \theta ={\left[\delta \theta_{x},\delta \theta_{y},\delta \theta_{z}\right]}^{T}$$

and the residual terms are defined as

$$e_{g}\left(\delta \theta \right)=\exp\left(\delta \theta^{\wedge }\right)R_{k}g_{imu}-g_{world}$$

$$e_{n}\left(\delta \theta \right)=\exp\left(\delta \theta^{\wedge }\right)R_{k}n_{rgbd}-n_{world}$$

$$e_{\psi }\left(\delta \theta \right)=\psi_{\mathrm{imu}}-\psi \left(\exp\left(\delta \theta^{\wedge }\right)R_{k}\right)$$

where $\psi_{\mathrm{imu}}$ denotes the short-term yaw angle obtained by integrating the IMU gyroscope measurements between adjacent frames, and ψ(·) extracts the yaw angle from the updated rotation matrix.

It should be noted that the yaw angle obtained by gyroscope integration is subject to accumulated drift over long-term operation. Therefore, the IMU-derived yaw constraint is not used as an absolute yaw reference in the proposed system. Instead, it serves as a short-term relative rotation prior between adjacent frames.

During long-term operation, yaw drift is compensated by LiDAR scan matching, visual feature constraints, loop-closure constraints, and backend graph optimization. In particular, when loop closure is detected, the accumulated heading error can be redistributed through global pose graph optimization, thereby reducing long-term yaw drift.

Therefore, the gyroscope-integrated yaw constraint mainly improves short-term rotation continuity, while long-term yaw consistency is maintained by multi-sensor geometric constraints and global optimization.

$W_{g}$ is the weight associated with the IMU measurement. When the IMU detects large non-gravitational acceleration, the reliability of the gravity measurement decreases and this weight is reduced.

$W_{n}$ is the weight associated with the RGB-D plane fitting result. When the confidence of the plane fitting is low or the RANSAC residual becomes large, the weight is also reduced.

The objective function is optimized using the Gauss–Newton algorithm. First, a first-order Taylor expansion is applied to linearize the residual around the current estimate.

The core objective of this optimization process is to reformulate the rotation estimation problem from Euclidean space onto the Lie group SO(3), thereby avoiding the singularities associated with conventional Euler angle representations. Moreover, by performing incremental updates in the corresponding Lie algebra, each iteration inherently preserves the orthogonality constraints of the rotation matrix, leading to improved numerical stability.

Since the rotation matrix must remain orthogonal, the update is performed on the Lie group using

(25) $$\delta R=\exp(\delta \theta^{\wedge })$$

and the rotation update becomes

(26) $$R_{k+1}=\delta R\cdot R_{k}$$

According to the chain rule, the derivative of the residual with respect to the perturbation δθ is

(27) $$\frac{\partial e}{\partial \delta \theta }=\frac{\partial (\left(\delta R\right)R_{k}v_{b})}{\partial \delta \theta }=\frac{\partial (\left(I+\delta \theta^{\wedge }\right)R_{k}v_{b})}{\partial \delta \theta }$$

The Jacobians of the residual terms are

(28) $$J_{g}=\frac{\partial e_{g}}{\partial \delta \theta }=-{\left(R_{k}g_{imu}\right)}^{\wedge }$$

(29) $$J_{n}=\frac{\partial e_{n}}{\partial \delta \theta }=-{\left(R_{k}n_{rgbd}\right)}^{\wedge }$$

(30) $$J_{\psi }=\frac{\partial e_{\psi }}{\partial \delta \theta }=\frac{\partial \psi (R_{k+1})}{\partial R_{k+1}}\frac{\partial R_{k+1}}{\partial \delta \theta }$$

Therefore, the Jacobian matrix of the entire objective function can be written as

(31) $$J=\left[\begin{array}{c} W_{g}\left(-{\left(R_{k}g_{imu}\right)}^{\wedge }\right)\\ W_{n}\left(-{\left(R_{k}n_{rgbd}\right)}^{\wedge }\right)\\ W_{\psi }J_{\psi } \end{array}\right]$$

The linear system is solved as

(32) $$\left(J^{T}J\right)\delta \theta =-J^{T}e$$

and the rotation estimate is updated as

(33) $$R_{k+1}=\exp\left(\delta \theta^{\wedge }\right)R_{k}$$

The iteration continues until the objective function converges, yielding the optimal rotation estimate. In summary, the primary role of the rotation estimation module in this section is to provide stable and high-accuracy attitude estimates, which are then fed back as known inputs into the LiDAR point cloud undistortion and the EKF-based translation update. This effectively blocks the propagation of rotational errors into the translational states. Such a decoupling strategy is crucial for improving the overall accuracy and robustness of the system.

### 4.4. Multi-Sensor Fusion SLAM Framework

The multi-sensor data are first preprocessed and temporally aligned to ensure consistent timestamps across different sensors. In the front-end, the translation and rotation components of the robot pose are estimated separately using the decoupled strategy described in Section 4.2 and Section 4.3. After obtaining the translation estimate from the EKF module and the rotation estimate from the Lie-group optimization module, the two components are synchronized based on their timestamps to form a complete 6-DoF pose representation. This pose serves as the initial estimate of the robot state in the global coordinate frame, together with the associated covariance matrix representing the uncertainty of the estimate.

However, since the front-end state update only considers constraints between consecutive frames, localization errors gradually accumulate over time due to sensor noise and motion uncertainty. To mitigate this issue, a back-end optimization module based on sliding-window factor graph optimization is introduced to further refine the robot state estimate.

Within the sliding window, several keyframes are maintained and constraints are established among them. These constraints include visual feature observations, IMU measurements, and wheel odometry information. During the factor graph optimization process, multiple types of factors are incorporated, including IMU factors, odometry factors, visual feature factors, scan-to-submap constraints, loop closure constraints, and the proposed roll angle smoothing factor. By jointly optimizing these factors, the system achieves improved global consistency and robustness.

#### 4.4.1. Back-End Optimization

Although the front-end decoupled estimation algorithm can provide high-frequency local pose estimates, errors inevitably accumulate over long-term operation due to sensor noise, IMU bias random walk, and subtle wheel slip. To address this issue, this study introduces a sliding-window-based factor graph optimization framework to impose global consistency constraints on the local trajectory and perform error correction.

The back-end optimization of SLAM is essentially a state estimation problem. Given a sequence of sensor observations *Z*, the objective is to estimate the system state *X* at different time steps. Within a probabilistic framework, the optimal state estimate can be obtained by maximizing the posterior probability P(X|Z).

According to Bayes’ rule,

(34) $$P\left(X|Z\right)=\frac{P(Z|X)P(X)}{P(Z)}$$

Since P(Z) is independent of the state *X*, maximizing the posterior probability is equivalent to maximizing the numerator:

(35) $$X_{MAP}^{*}=\arg\max_{X}P\left(X|Z\right)\propto \arg\max_{X}P\left(Z|X\right)P\left(X\right)$$

Here, P(Z|X) represents the likelihood, describing the probability of obtaining observation *Z* under state *X*, which is defined by the sensor observation model. P(X) denotes the prior distribution, representing prior knowledge or assumptions about the state.

Assuming that the sensor observation noise and motion model noise are independent and follow Gaussian distributions, consider a measurement $z_{k}$ associated with states $x_{i}$ and $x_{j}$. The error term is defined as

$$e_{k}\left(x_{i},x_{j}\right)=z_{k}-h_{k}\left(x_{i},x_{j}\right)$$

which follows a zero-mean Gaussian distribution

$$N(0,\Sigma_{k})$$

Therefore, the likelihood can be written as

(36) $$P\left(z_{k}|x_{i},x_{j}\right)\propto \exp\left(-\frac{1}{2}{∥h_{k}\left(x_{i},x_{j}\right)-z_{k}∥}_{\Sigma_{k}}^{2}\right)$$

where the squared Mahalanobis norm is defined as

$${∥e∥}_{\Sigma }^{2}=e^{T}\Sigma^{-1}e$$

By incorporating all observations and prior information, the maximum a posteriori estimation can be converted into a nonlinear least-squares problem:

(37) $$X^{*}=\arg\min_{X}\left(\sum_{k}∥e_{k}\left(x_{i},x_{j}\right){∥}_{\Sigma_{k}}^{2}+{∥e_{prior}\left(x_{0}\right)∥}_{\Sigma_{prior}}^{2}\right)$$

The factor graph provides an intuitive representation of this optimization problem. The entire graph consists of nodes and factors. Each node stores a system state at a particular time, typically representing the robot pose in the world coordinate frame. Factors represent constraints between nodes, which correspond to sensor measurements.

**Figure 3:**
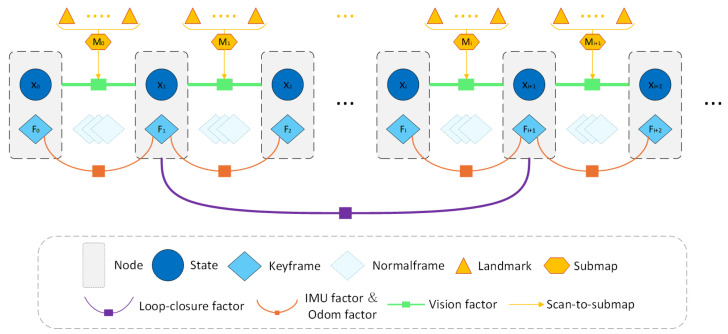
Factor graph representation of the back-end optimization in ROIV-SLAM. The nodes represent system states associated with keyframes, while different factors encode constraints from multiple sensors, including IMU preintegration, wheel odometry, visual observations, scan-to-submap matching, and loop closure. The proposed roll angle smoothing constraint improves the stability of rotation estimation under high-frequency roll disturbances.

In this work, multiple types of constraints are incorporated into the factor graph, including IMU preintegration factors, wheel odometry factors, LiDAR scan matching factors, visual feature factors, loop closure factors, and the proposed roll angle smoothing factor. These factors constrain the relative transformation between adjacent states, forming a chain-like structure similar to a Markov chain. The relative pose transformation between nodes is estimated through scan-to-map matching. The factor graph structure used in the back-end optimization is shown in Figure 3.

Within this framework, observations from different sensors are uniformly modeled as factors and fused within a unified probabilistic graph optimization framework. In this manner, the complementary characteristics of multiple sensors can be fully exploited, effectively suppressing the errors of individual sensors while improving the overall estimation accuracy.

IMU provides high-frequency acceleration and angular velocity measurements. Direct integration of these measurements would lead to significant drift over time. To address this issue, the IMU preintegration technique aggregates all IMU measurements between two consecutive keyframes into a single relative motion constraint. This approach effectively handles high-frequency measurements while modeling IMU noise and bias.

In the factor graph formulation, the IMU preintegration factor connects two consecutive keyframe states $X_{i}$ and $X_{j}$ together with the corresponding IMU bias terms $B_{i}$ and $B_{j}$. The residual term is constructed from the difference between the predicted motion obtained through preintegration and the actual states, including rotation, position, and velocity components.

The IMU residual can be written as

(38) $$e_{imu}=\left[\begin{array}{c} \log\left({\left(\Delta R_{ij}\right)}^{T}R_{i}^{T}R_{j}\right)\\ R_{i}^{T}\left(P_{j}-P_{i}-V_{i}\Delta t\right)-\Delta P_{ij}\\ R_{i}^{T}\left(V_{j}-V_{i}\right)-\Delta V_{ij}\\ B_{j}-B_{i} \end{array}\right]$$

The LiDAR scan-matching factor estimates the relative pose transformation between the current keyframe scan and the local map using the ICP algorithm. This constraint is used to correct the accumulated drift from IMU and wheel odometry.

Since the front-end has already jointly optimized the gravity vector from the IMU and the ground normal extracted from RGB-D observations to estimate rotation, the visual feature matching constraint between keyframes can be obtained by matching feature points between two keyframes. The relative pose $\Delta T_{visual}$ is then estimated using the PnP-RANSAC algorithm with depth information.

To further improve coupling between visual and geometric constraints, 3D map points are directly introduced as optimization variables. For a map point with 3D coordinates $P_{w}$ observed in keyframe $X_{i}$, its projection in keyframe $X_{j}$ is

$$\pi (T_{j}^{-1}P_{w})$$

The corresponding visual residual is defined as

(39) $$e_{visual}=z_{j}-\pi \left(T_{j}^{-1}P_{w}\right)$$

where $z_{j}$ denotes the observed pixel coordinates of the map point in the image.

##### Roll Angle Smoothing Factor

Most existing VIO or LIO methods usually assume that the robot moves on a flat ground surface, where the roll angle variation is relatively small. However, the non-coaxial self-balancing two-wheeled robot considered in this work is a typical inverted-pendulum-like system. During forward motion, steering, or motion over uneven ground, the robot relies on the momentum wheel to perform high-frequency attitude regulation for maintaining lateral balance. Therefore, the roll angle of the robot does not remain constant during SLAM operation, but continuously exhibits high-frequency small- or medium-amplitude fluctuations.

If the pose estimation relies only on conventional frame-to-frame relative matching, the optimizer may be easily affected by abnormal observations when environmental features are sparse, visual measurements are degraded, or sensor data quality decreases. This may further lead to non-physical attitude jumps. To address this issue, a roll angle smoothing factor is introduced into the back-end factor graph to constrain the higher-order temporal continuity of the roll angle between adjacent keyframes.

Let three consecutive keyframes in the sliding window be denoted as i−1, *i*, and i+1, with timestamps $t_{i-1}$, $t_{i}$, and $t_{i+1}$, respectively. The corresponding roll angles extracted from the rotation matrices of the state nodes are denoted as $\alpha_{i-1}$, $\alpha_{i}$, and $\alpha_{i+1}$. When the time interval between adjacent keyframes is sufficiently short, the roll angular velocity of the robot can be assumed to vary smoothly, which gives

(40) $$\frac{\alpha_{i+1}-\alpha_{i}}{t_{i+1}-t_{i}}\approx \frac{\alpha_{i}-\alpha_{i-1}}{t_{i}-t_{i-1}}.$$

When the keyframe time intervals are approximately equal, namely

(41) $$t_{i+1}-t_{i}\approx t_{i}-t_{i-1}=\Delta t,$$

the above relationship can be further written as a second-order difference constraint on the roll angle:

(42) $$\left(\alpha_{i+1}-\alpha_{i}\right)-\left(\alpha_{i}-\alpha_{i-1}\right)\approx 0.$$

Therefore, the residual function of the roll angle smoothing factor is defined as

(43) $$e_{\mathrm{smooth}}\left(X_{i-1},X_{i},X_{i+1}\right)=\alpha_{i+1}-2\alpha_{i}+\alpha_{i-1}.$$

After this residual is introduced into the back-end factor graph optimization, the corresponding cost term is formulated as

(44) $$J_{\mathrm{smooth}}=\frac{1}{2}{∥e_{\mathrm{smooth}}∥}_{\Sigma_{\mathrm{smooth}}^{-1}}^{2},$$

where $\Sigma_{\mathrm{smooth}}$ denotes the covariance of the roll angle smoothing factor and controls the weight of this constraint in the overall optimization objective.

Unlike purely empirical parameter tuning, the covariance in this work is determined according to the physical roll-dynamic boundary of the non-coaxial self-balancing two-wheeled robot. For this type of robot, the roll angle variation is mainly determined by the reaction torque generated by the momentum wheel, the gravity-induced restoring torque, and external disturbance torque. The roll dynamics around the longitudinal axis of the robot can be written as

(45) $$J_{x}\ddot{\alpha }=\tau_{\mathrm{wheel}}+mgL\sin\alpha -\tau_{\mathrm{disturb}},$$

where $J_{x}$ is the moment of inertia of the robot body around the roll axis, *m* is the body mass, *L* is the height of the center of mass, $\tau_{\mathrm{wheel}}$ is the torque generated by the momentum wheel, and $\tau_{\mathrm{disturb}}$ denotes the external disturbance torque.

In practice, the covariance $\Sigma_{\mathrm{smooth}}$ was selected according to the expected physical range of roll angle fluctuations observed during robot balancing. A relatively small covariance increases the contribution of the smoothing factor and suppresses high-frequency oscillatory roll motion, whereas an excessively small covariance may over-constrain the optimization and suppress physically valid rotational dynamics. Conversely, assigning an excessively large covariance weakens the smoothing effect and reduces robustness against balancing-induced rotational disturbances.

Similarly, the weighting parameters associated with the gravity and ground-normal constraints were empirically selected according to the sensor noise characteristics and environmental stability. In the experiments reported in this work, a single fixed parameter configuration was used across all tested environments without scene-specific retuning. Stable localization performance under different operating conditions suggests that the proposed framework maintains reasonable robustness with respect to parameter selection.

Considering the limited output capability of the motor, the maximum instantaneous peak torque generated by the momentum wheel is denoted as $\tau_{\max}$. Without considering extreme external impacts, the roll angular acceleration of the robot is physically bounded by

(46) $$|\ddot{\alpha }|\leq {\ddot{\alpha }}_{\max}\approx \frac{\tau_{\max}+mgL}{J_{x}}.$$

In the discrete SLAM sliding window, when the adjacent keyframe interval is Δt, the second-order central difference of the roll angle can be approximated as

(47) $${\ddot{\alpha }}_{i}\approx \frac{\alpha_{i+1}-2\alpha_{i}+\alpha_{i-1}}{\Delta t^{2}}.$$

Therefore, the roll angles of three consecutive keyframes should satisfy the following physical boundary:

(48) $$\left|\frac{\alpha_{i+1}-2\alpha_{i}+\alpha_{i-1}}{\Delta t^{2}}\right|\leq {\ddot{\alpha }}_{\max}.$$

This further leads to

(49) $$\left|\alpha_{i+1}-2\alpha_{i}+\alpha_{i-1}\right|\leq {\ddot{\alpha }}_{\max}\Delta t^{2}.$$

The above inequality indicates that the roll angle smoothing residual $e_{\mathrm{smooth}}$ has a clear physically feasible range. To ensure that the smoothing factor can suppress non-physical attitude jumps while avoiding excessive suppression of real roll motion, the standard deviation of the smoothing factor is defined as a scaled form of the physical boundary of the second-order roll angle variation:

(50) $$\sigma_{\mathrm{smooth}}=\kappa \cdot {\ddot{\alpha }}_{\max}\cdot \Delta t^{2}=\kappa \cdot \frac{\tau_{\max}+mgL}{J_{x}}\cdot \Delta t^{2}.$$

The corresponding covariance is then given by

(51) $$\Sigma_{\mathrm{smooth}}=\sigma_{\mathrm{smooth}}^{2}.$$

Here, κ is a confidence scaling coefficient that adjusts the relationship between the physical boundary and the optimization weight. In general, κ can be selected within the range of 0.8 to 1.2. A smaller κ increases the weight of the smoothing constraint, making the optimizer more inclined to suppress abrupt roll angle changes. In contrast, a larger κ weakens the smoothing constraint and allows the optimizer to respond more flexibly to rapid real roll motion. In this work, the same value of κ is used in all experiments to ensure parameter consistency across different environments and disturbance conditions.

From the perspective of robust optimization, this smoothing factor can be regarded as a second-order Markov smoothness constraint with a physically adaptive boundary. When sensor mismatch or local observation degradation causes an abnormal roll estimate at a certain keyframe, the corresponding residual $|e_{\mathrm{smooth}}|$ may exceed the physically reasonable range characterized by $\sigma_{\mathrm{smooth}}$. In this case, the abnormal attitude estimate is penalized during optimization and pulled back toward a trajectory that is more consistent with the inverted-pendulum-like roll dynamics of the robot. Therefore, the proposed factor suppresses roll angle error leakage caused by motion degradation and improves the attitude continuity and overall trajectory stability of the backend optimization.

In practice, the proposed covariance design exhibits relatively low sensitivity to moderate variations of the scaling coefficient κ. Since the covariance is derived from physically bounded roll dynamics rather than purely empirical tuning, changing κ within a reasonable range mainly affects the strength of the smoothing constraint without altering the overall optimization behavior. Experimental observations indicate that the proposed system maintains stable trajectory consistency when κ varies within the range of 0.8 to 1.2. Therefore, the proposed formulation provides a physically interpretable and robust mechanism for determining the covariance of the roll angle smoothing factor.

In addition, loop closure detection is introduced to eliminate long-term drift. When the robot revisits a previously mapped area, loop closure detection identifies this event and establishes a strong constraint between the current pose node and historical pose nodes. This constraint pulls the trajectory back to the correct position, resulting in a globally consistent map.

As time progresses, the size of the factor graph grows continuously, which may lead to computational explosion. To maintain real-time performance, a sliding-window optimization strategy is adopted, where only the most recent *N* keyframes are retained for optimization.

When the window becomes full and a new keyframe is added, the oldest keyframe is marginalized. Marginalization compresses all constraints associated with the removed keyframe into a prior factor and propagates the uncertainty to the remaining variables. This prior factor preserves the information contained in the marginalized keyframe while keeping the optimization problem size manageable.

#### 4.4.2. Loop Closure Detection

Loop closure detection plays an important role in SLAM systems. Without loop closure constraints, the robot trajectory gradually accumulates drift over time due to sensor noise and estimation errors. When the robot revisits a previously mapped area, loop closure detection identifies this event and introduces additional constraints to correct the accumulated error, thereby improving the global consistency of the map.

In this work, a vision-based place recognition method is adopted to detect loop closure candidates. Recent RGB-D and visual SLAM studies in dynamic indoor environments have shown that dynamic feature suppression and robust visual front-end design are important for maintaining reliable loop detection and pose estimation under degraded visual observations [25]. Specifically, a Bag-of-Words (BoW) model is constructed using visual features extracted from RGB images. Each keyframe image is converted into a visual word vector, which enables efficient similarity search among previously observed keyframes.

Let the visual word vectors of two keyframes *i* and *j* be denoted as $v_{i}$ and $v_{j}$, respectively. The similarity between these two vectors is measured using cosine similarity:

(52) $$s_{ij}=\frac{v_{i}\cdot v_{j}}{∥v_{i}∥∥v_{j}∥}$$

When the similarity score exceeds a predefined threshold, the corresponding keyframe pair is considered a loop closure candidate.

To verify the geometric consistency of the detected loop, feature correspondences between the two images are established and the relative pose transformation is estimated using the PnP-RANSAC algorithm. If the number of inlier correspondences exceeds a threshold, the loop closure is accepted.

After the loop closure candidate is verified, LiDAR scan matching is further performed between the corresponding scans to refine the relative pose transformation. The loop-closure residual is formulated on the Lie group manifold as

(53) $$e_{loop}=\log\left({\left(T_{ij}^{obs}\right)}^{-1}T_{i}^{-1}T_{j}\right)$$

where log(·) denotes the logarithmic map from the Lie group SE(3) to its corresponding Lie algebra representation.

The loop closure factor is then added into the factor graph optimization framework, which effectively reduces accumulated drift and improves the global consistency of the estimated trajectory and map. In summary, the primary role of the back-end optimization module is to enforce global consistency on the front-end estimation results. By fusing multi-source constraints, it effectively suppresses error accumulation, thereby ensuring the accuracy and stability of the system during long-term operation.

## 5. Experimental Results

### 5.1. Baseline Selection Rationale

To ensure a fair comparison, all evaluated methods were required to operate using the same sensor configuration available on the experimental platform.

The robot is equipped with a 2D LiDAR, an RGB-D camera, an IMU, and wheel odometry. Consequently, representative SLAM systems designed for 3D LiDAR sensors, including LIO-SAM, FAST-LIO2, LVI-SAM, and R3LIVE, were not directly applicable without modifying the sensor hardware or generating artificial 3D point clouds from planar scans.

Such modifications would introduce additional uncertainties and compromise comparison fairness.

Therefore, the evaluation focuses on representative methods that can operate under the same sensing conditions, including Cartographer and RTAB-Map.

Cartographer represents a mature LiDAR-based SLAM framework, whereas RTAB-Map represents a widely adopted vision-based SLAM framework.

The proposed ROIV-SLAM can thus be evaluated against both sensing modalities under identical hardware constraints.

### 5.2. Experimental Platform

**Figure 4:**
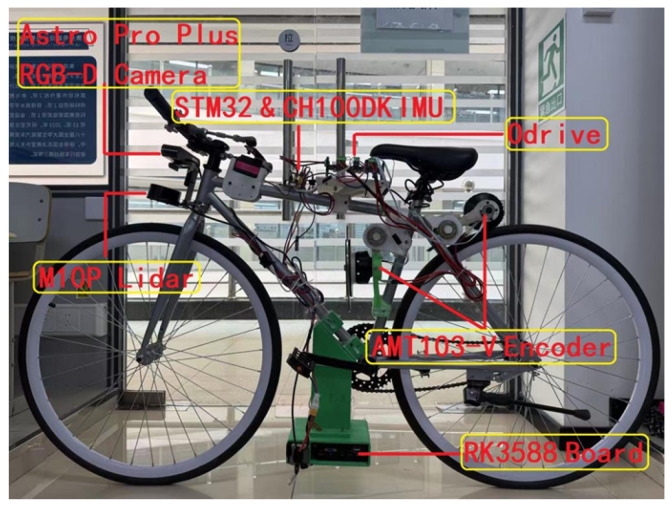
Hardware configuration of the experimental platform.

The experimental platform is built upon a self-developed non-coaxial two-wheeled robotic system, which consists of a perception-computation module and a low-level control module. As shown in Figure 4, the perception and computing unit is based on the RK3588 embedded platform, featuring an octa-core heterogeneous architecture that ensures efficient multi-thread processing and real-time performance for multi-sensor data fusion. The system is equipped with an RGB-D camera for capturing both color and depth information, along with a 2D LiDAR for acquiring geometric structure of the environment.

To provide sufficient implementation details, the main sensor specifications and calibration procedures are summarized as follows. The experimental platform is equipped with an RK3588-based embedded computing platform manufactured by Rockchip Electronics Co., Ltd. (Fuzhou, China), a CH100DK IMU manufactured by Beijing Chaonuclear Electronic Co., Ltd. (Beijing, China), a Leishen M10P 2D LiDAR manufactured by Shenzhen Leishen Intelligent System Co., Ltd. (Shenzhen, China), and an Orbbec Astra Pro Plus RGB-D camera manufactured by Orbbec Co., Ltd. (Shenzhen, China). The IMU operates at 200 Hz and provides three-axis angular velocity and linear acceleration measurements. The 2D LiDAR provides 360-degree planar scans at 12 Hz with a maximum measurement range of 30 m and a sampling rate of 20,000 points/s. The RGB-D camera operates at 30 Hz, with an RGB resolution of 1920 × 1080 and a depth resolution of 640 × 480. Its effective depth range is approximately 0.6–8 m, with a horizontal field of view of 58.4° and a vertical field of view of 45.5°.

The intrinsic parameters of the RGB-D camera were calibrated offline using a standard checkerboard-based calibration procedure. The spatial extrinsic parameters among the IMU, 2D LiDAR, RGB-D camera, and robot body frame were also calibrated offline. Specifically, the rigid transformations between sensors were represented as homogeneous transformation matrices and were used to project measurements into a unified robot coordinate frame during state estimation and back-end optimization.

Time synchronization was performed using the IMU timestamp as the main temporal reference. Since the IMU has the highest sampling rate, its measurements were used for high-frequency state propagation. The lower-rate LiDAR and RGB-D measurements were then associated with the nearest IMU timestamp and used as observation updates. This synchronization strategy reduces the influence of asynchronous sensor measurements and improves the consistency of multi-sensor fusion under high-frequency roll disturbances.

This platform provides a challenging testbed with continuous roll disturbances, making it suitable for evaluating the robustness of the proposed ROIV-SLAM method.

Since no external motion-capture system or high-precision positioning infrastructure was available in the experimental environment, a reference trajectory was generated through offline global optimization using the complete dataset collected during the experiment. The reference trajectory was generated using full-sequence offline optimization with manually verified loop closures and globally consistent pose refinement. Although this trajectory does not constitute an independent ground-truth measurement obtained from external positioning systems, it provides a highly consistent reference for comparative evaluation under identical sensing conditions. Therefore, the reported APE values should be interpreted as relative trajectory consistency errors rather than absolute positioning errors. This reference trajectory was subsequently used for trajectory comparison and quantitative error evaluation.

### 5.3. Mapping Performance and Trajectory Consistency Analysis

**Figure 5:**
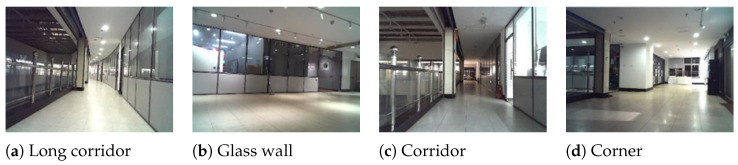
Experimental scenario.

To comprehensively evaluate the global consistency and mapping accuracy of the proposed system, experiments were conducted in a looped indoor environment consisting of long corridors and glass curtain walls (see Figure 5). The robot traversed the entire loop once, while three different methods were tested in parallel, including a LiDAR-only SLAM (Cartographer 2D variant) [26], a vision-only SLAM [27], and the proposed multi-sensor fusion method ROIV-SLAM.

#### 5.3.1. Global Map Topology and Ghosting Analysis

**Figure 6:**
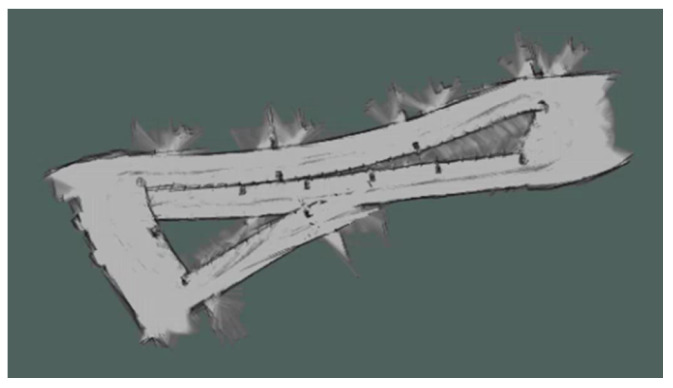
LiDAR mapping.

**Figure 7:**
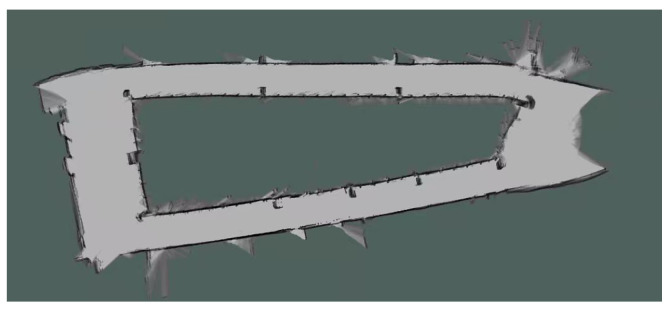
RGB-D mapping.

**Figure 8:**
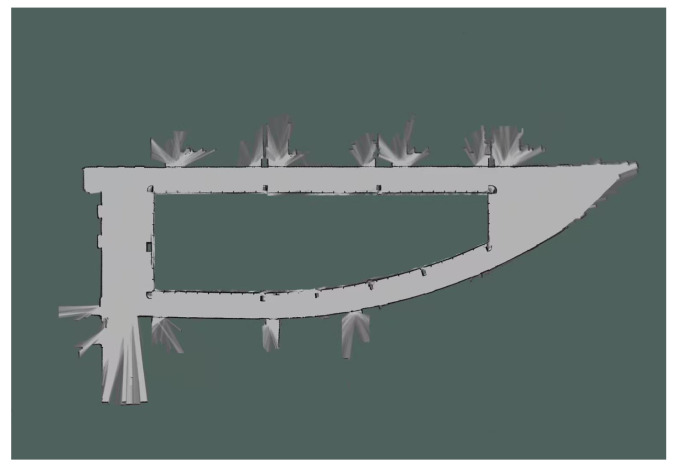
Fusion mapping.

The generated global occupancy grid maps are shown in Figure 6, Figure 7 and Figure 8, where significant differences can be observed.

As shown in Figure 6, the LiDAR-only mapping suffers from severe distortion. During turning motions, large steering angles and roll disturbances cause the 2D LiDAR scanning plane to deviate from the horizontal plane. In corridor environments with limited longitudinal features, such deviations introduce pseudo-observations, leading to failure in scan matching and pose tracking. In the loop closure region, this results in misalignment and ghosting artifacts, ultimately producing a warped map that is unsuitable for global path planning.

The RGB-D mapping results (Figure 7) also exhibit several limitations. The camera has a restricted field of view and limited sensing range. During rapid motion and turning, high-frequency disturbances introduce motion blur, which degrades feature quality and increases scale drift in visual odometry. Although loop closure is achieved, unreliable feature matching and strong reliance on IMU and wheel odometry lead to accumulated errors. When loop closure constraints are enforced, the map tends to be artificially deformed, resulting in noticeable scale inconsistencies with the real environment.

**Figure 9:**
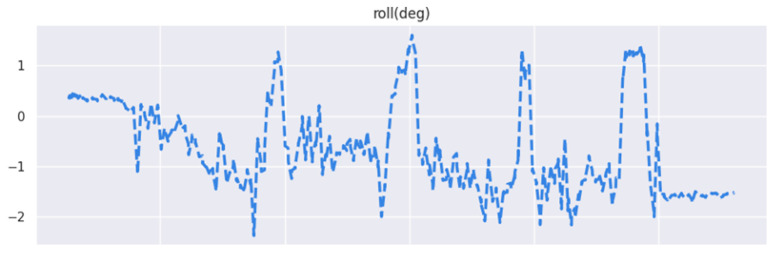
Variation of the roll angle during the experiment.

In contrast, the proposed ROIV-SLAM (Figure 8) produces a globally consistent map. Benefiting from the roll angle smoothing factor introduced in the sliding-window backend, the system effectively suppresses distortion caused by abnormal poses. Moreover, the combination of visual loop detection and LiDAR geometric verification ensures robust loop closure. The bag-of-words model enables long-range place recognition, while geometric refinement compensates for the limitations of visual pose estimation. As a result, the reconstructed map preserves clear structural boundaries without ghosting artifacts, successfully recovering the looped corridor layout. Minor deviations are observed in curved sections due to dynamic motion, and slight noise appears near reflective glass surfaces; however, the overall global consistency is well maintained. The corresponding roll angle variation during the experiment is shown in Figure 9.

#### 5.3.2. Trajectory Comparison

**Figure 10:**
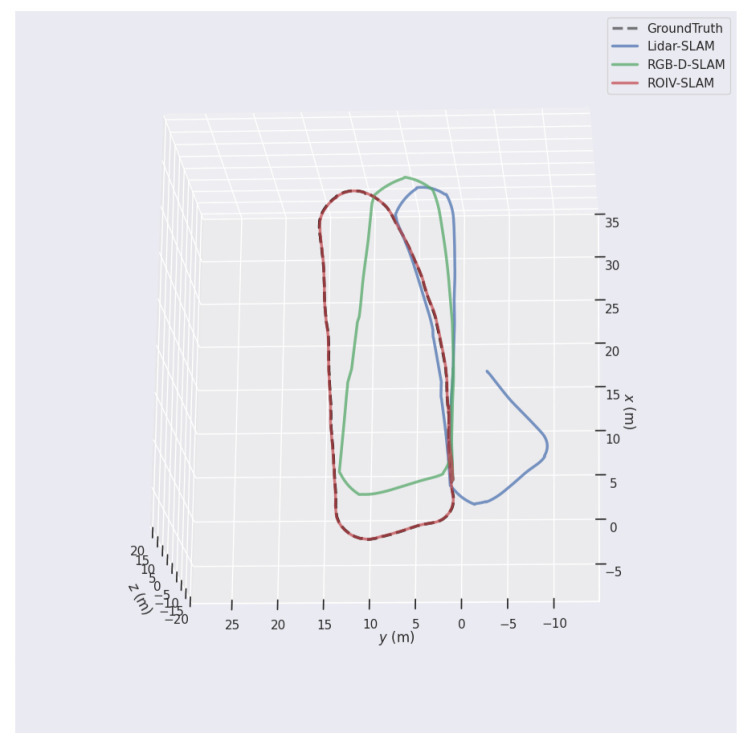
Trajectory output comparison.

**Figure 11:**
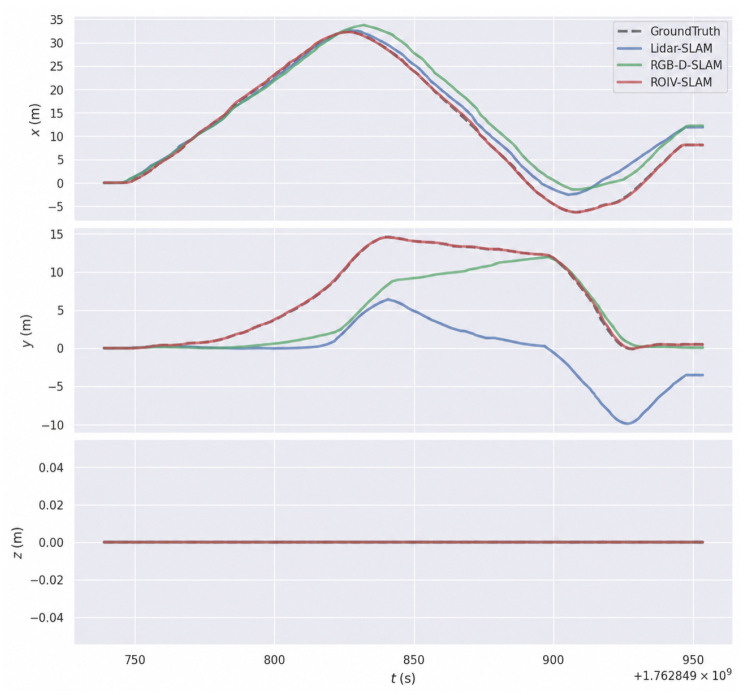
Trajectory deviation.

The optimized trajectories from the three methods are compared with the reference trajectory generated through offline global optimization using the complete experimental dataset. The 3D trajectory comparison is shown in Figure 10, and the axis-wise trajectory deviations are illustrated in Figure 11.

The results show that the ROIV-SLAM trajectory (red curve) closely aligns with the reference trajectory, especially in the horizontal plane, where the estimated displacement matches well with the reference trajectory. In contrast, the LiDAR-only trajectory (blue curve) exhibits significant accumulated drift, particularly along the *y*-axis after approximately 5 m of motion along the *x*-axis. The drift becomes more pronounced after turning, eventually leading to physically inconsistent deviations. Although partial correction occurs near the final segment, the trajectory fails to close properly, with overall errors of approximately 5 m.

The vision-only trajectory achieves loop closure; however, its orientation deviates significantly from the reference trajectory. Due to limited field of view and motion disturbances, feature association degrades during turning, resulting in yaw estimation errors. This leads to increasing deviation in the *y*-axis after the second turn, and although the final *y* displacement aligns with the reference trajectory, the *x*-axis still exhibits an error of approximately 5 m.

#### 5.3.3. APE-Based Trajectory Consistency Analysis

**Figure 12:**
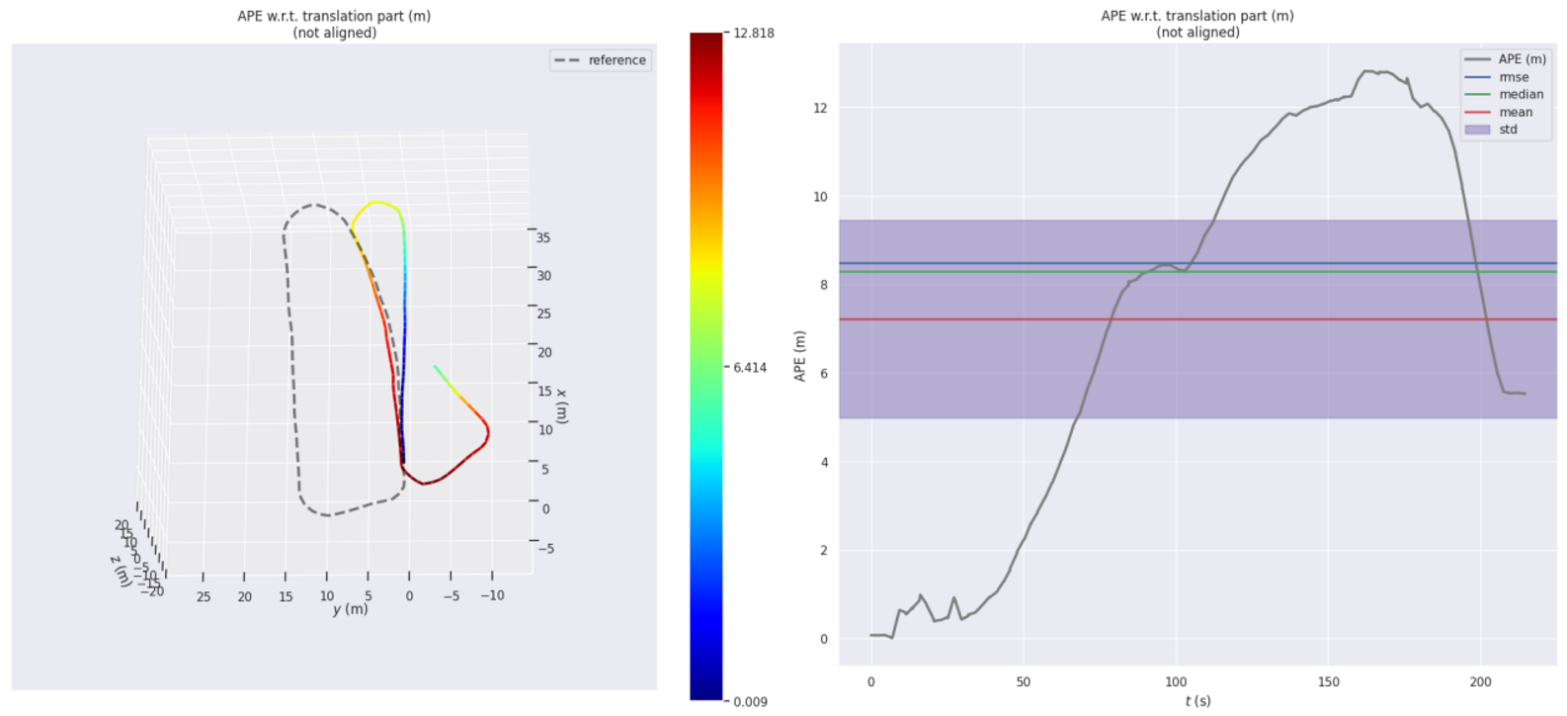
APE with respect to the optimized reference trajectory for LiDAR SLAM.

**Figure 13:**
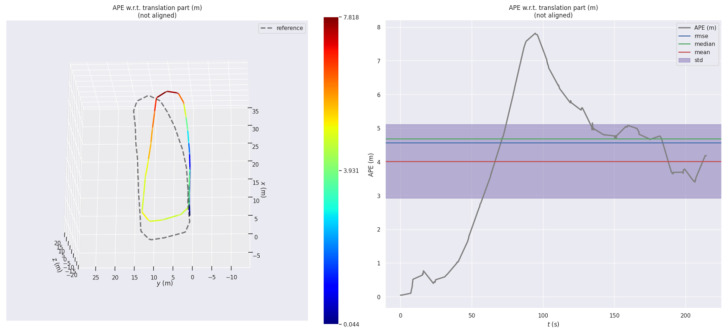
APE with respect to the optimized reference trajectory for RGB-D SLAM.

**Figure 14:**
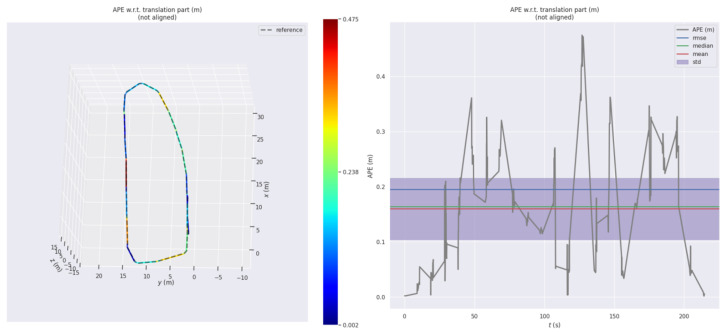
APE with respect to the optimized reference trajectory for ROIV-SLAM.

The APE results for LiDAR SLAM, RGB-D SLAM, and ROIV-SLAM are shown in Figure 12, Figure 13, and Figure 14, respectively, along with the quantitative comparison summarized in Table 1.

As summarized in Table 1, the LiDAR SLAM and RGB-D SLAM baselines yield mean APE values of 7.21 m and 4.01 m, respectively, with RMSE values of 8.48 m and 4.57 m. Since the reference trajectory is generated through offline global optimization rather than an independent external ground-truth system, these APE values should be interpreted as trajectory consistency errors with respect to the optimized reference trajectory.

Under this evaluation setting, ROIV-SLAM achieves a mean APE of 0.15 m and an RMSE of 0.19 m, indicating better consistency with the optimized reference trajectory than the evaluated baseline methods in the tested scenario. In addition, its standard deviation is 0.11 m, suggesting a more concentrated error distribution under the tested high-frequency roll disturbance conditions. These results indicate that the proposed framework, which integrates platform dynamics priors with multimodal factor-graph optimization, improves trajectory consistency and mapping robustness under the evaluated sensing configuration.

To further evaluate computational cost and efficiency, the runtime and memory consumption of different methods are also compared in Table 1. In terms of runtime, ROIV-SLAM requires 6.78% more time than LiDAR SLAM, while being 4.84% faster than RGB-D SLAM. Regarding memory usage, ROIV-SLAM consumes 60.71% more memory than LiDAR SLAM and 7.14% more than RGB-D SLAM. Although the proposed fusion framework introduces additional computational overhead, it provides better trajectory consistency with respect to the optimized reference trajectory and produces more coherent maps than the evaluated baselines in the tested scenario.

For the current industrial computer equipped with 8GB memory, the additional resource consumption remains within an acceptable range, accounting for less than 10% of the available memory. The increased cost mainly arises from the need to process both LiDAR and visual point cloud data simultaneously, while the back-end and loop-closure threads perform feature extraction, feature matching, and bag-of-words-based place recognition. In addition, the system maintains sliding-window states, Schur-complement-based marginalization priors, and concurrent processing of both visual and LiDAR submaps. From an engineering perspective, this additional computational cost is acceptable considering the improved trajectory consistency and map coherence observed in the tested scenarios. Therefore, the proposed method provides a practical solution for the target lightweight robotic platform.

#### 5.3.4. Real-Time Performance Analysis

While Table 1 reports the overall runtime and memory consumption of different SLAM systems, Table 2 further analyzes the execution time of individual modules within the proposed ROIV-SLAM framework.

As shown in Table 2, the front-end sensor processing and state estimation modules require only 24.8 ms and 11.6 ms on average, respectively. The additional roll angle optimization introduces limited computational overhead and therefore has little impact on real-time operation.

The major computational burden originates from loop-closure detection and back-end graph optimization, which require 31.4 ms and 42.7 ms, respectively. However, these modules are executed asynchronously in separate threads and therefore do not block the front-end tracking process.

The results indicate that the proposed ROIV-SLAM framework maintains efficient online operation while providing substantial improvements in trajectory consistency and global consistency. Therefore, the computational cost introduced by multimodal fusion and graph optimization remains acceptable for practical deployment on embedded robotic platforms.

### 5.4. Generalization and Robustness Evaluation

#### 5.4.1. Experimental Environments

To further evaluate the generalizability of the proposed ROIV-SLAM framework, experiments were conducted in two different indoor environments.

The first environment was the looped-corridor scenario used in the previous experiments. This environment contains a closed-loop trajectory with relatively regular corridor structures, making it suitable for evaluating loop-closure performance and accumulated drift suppression.

The second environment was a composite indoor scenario consisting of an L-shaped corridor and an obstacle-rich hall. Compared with the looped-corridor environment, this scenario contains more diverse spatial structures, including narrow passages, sharp turns, open spaces, and multiple obstacles. The L-shaped corridor introduces significant heading changes without relying on a simple closed-loop trajectory, while the obstacle-rich hall contains more complex visual and geometric features, partial occlusions, and locally irregular layouts.

**Figure 15:**
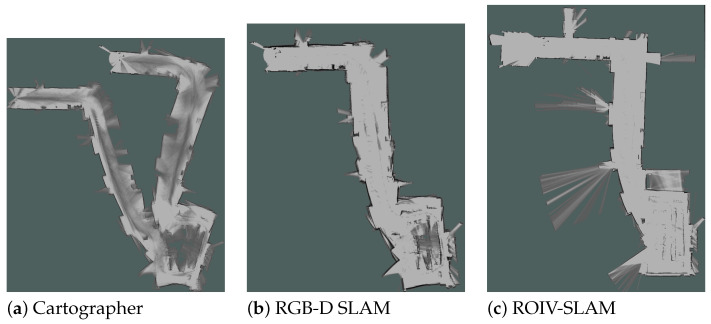
Comparison of mapping results generated by Cartographer, RGB-D SLAM, and the proposed ROIV-SLAM framework in the composite indoor environment consisting of an L-shaped corridor and an obstacle-rich hall. The proposed method produces a more geometrically consistent map with fewer structural distortions, demonstrating improved robustness in complex indoor scenarios.

Figure 15 presents the mapping results obtained by Cartographer, RGB-D SLAM, and the proposed ROIV-SLAM framework in the composite indoor environment. The comparison provides a qualitative evaluation of the mapping quality and structural consistency achieved by the three methods under more challenging indoor conditions. Although all methods are capable of reconstructing the main environmental structures, the map generated by ROIV-SLAM exhibits improved geometric consistency and reduced distortion in regions containing sharp turns and dense obstacles, indicating enhanced robustness against localization drift and motion disturbances.

To further evaluate the repeatability and localization consistency of the three SLAM methods, each algorithm was independently executed three times in the composite indoor environment. The estimated trajectories obtained from the repeated experiments were compared with the corresponding reference trajectory generated from the offline globally optimized map.

**Figure 16:**
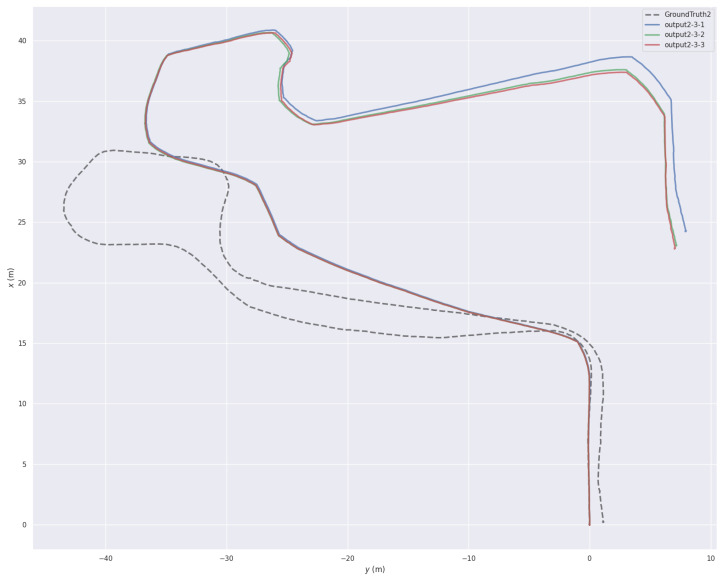
Trajectory comparison between three independent Cartographer runs and the reference trajectory in the composite indoor environment. Noticeable deviations can be observed among repeated experiments, indicating accumulated localization errors and reduced repeatability.

**Figure 17:**
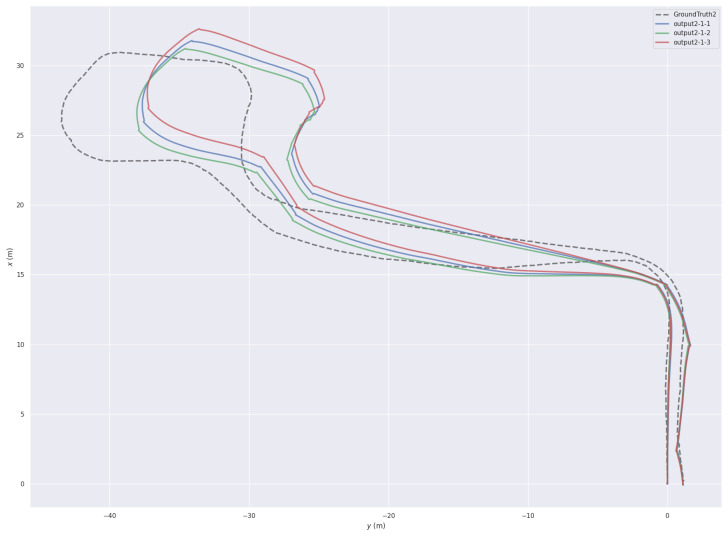
Trajectory comparison between three independent RGB-D SLAM runs and the reference trajectory in the composite indoor environment. Although the estimated trajectories generally follow the reference path, visible discrepancies remain in several sections of the trajectory.

**Figure 18:**
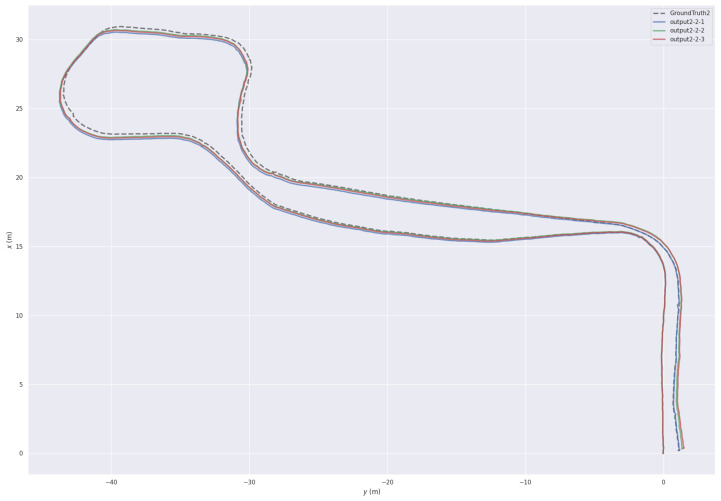
Trajectory comparison between three independent ROIV-SLAM runs and the reference trajectory in the composite indoor environment. The trajectories exhibit high consistency and closely match the reference trajectory, demonstrating improved localization stability and repeatability.

Figure 16, Figure 17 and Figure 18 show the trajectory comparisons for Cartographer, RGB-D SLAM, and ROIV-SLAM, respectively. For each algorithm, the trajectories from three independent runs are plotted together with the reference trajectory. The comparison allows qualitative assessment of trajectory consistency, accumulated drift, and sensitivity to environmental disturbances.

As shown in Figure 16, Cartographer exhibits noticeable trajectory deviations among different runs, particularly in regions involving sharp turns and long corridor segments. These deviations indicate the presence of accumulated localization errors and reduced repeatability under complex indoor conditions.

Figure 17 shows that RGB-D SLAM achieves better consistency than Cartographer; however, visible discrepancies between repeated runs still exist in several sections of the trajectory. The performance is influenced by variations in visual feature quality, viewpoint changes, and temporary occlusions.

In contrast, the trajectories generated by the proposed ROIV-SLAM framework in Figure 18 show greater overlap with the optimized reference trajectory and smaller variation among repeated experiments than the evaluated baseline methods. This result suggests improved trajectory repeatability and robustness against motion disturbances under the tested conditions, leading to more consistent trajectory estimation across multiple runs.

#### 5.4.2. Repeated-Trial Evaluation

To reduce the influence of random experimental variations and improve the statistical reliability of the evaluation, repeated trials were conducted for all compared methods in both experimental environments.

For each environment, three repeated trials were performed under the same hardware configuration and similar motion routes. The localization performance was evaluated using the Absolute Pose Error (APE), and the mean value and standard deviation over repeated runs were calculated.

The mean APE reflects the overall trajectory consistency, whereas the standard deviation indicates the repeatability and stability of each method across multiple runs.

As shown in Table 3, ROIV-SLAM achieves lower average APE than the compared methods in both the looped-corridor environment and the composite environment. Moreover, ROIV-SLAM exhibits a smaller standard deviation over repeated trials, indicating better repeatability and stability.

The performance improvement is particularly evident in the composite environment, where the robot traverses both narrow corridor segments and an obstacle-rich hall. These results suggest that the proposed framework is not limited to a single corridor scenario and maintains robust trajectory consistency under more diverse indoor conditions.

#### 5.4.3. Evaluation Under Different Roll-Disturbance Conditions

Since the proposed method is specifically designed for non-coaxial self-balancing two-wheeled robots operating under roll disturbances, additional experiments were conducted under different disturbance conditions.

The normal-disturbance condition corresponds to regular robot motion without deliberate external interference. The high-disturbance condition was generated by applying deliberate manual interference during robot motion, producing rapid and large-amplitude roll angle variations.

The roll angle statistics under different disturbance conditions are summarized in Table 4.

The localization performance under different roll-disturbance conditions is summarized in Table 5.

As shown in Table 5, increasing roll disturbance leads to performance degradation for all methods. However, the degradation of ROIV-SLAM is considerably smaller than that of the compared methods.

Under high-disturbance conditions induced by external manual interference, ROIV-SLAM still maintains stable trajectory estimation, whereas the compared methods exhibit more severe trajectory distortion and accumulated drift. Consequently, the reported Absolute Pose Error (APE) values should be interpreted as measures of trajectory consistency with respect to the optimized reference trajectory rather than absolute localization accuracy.

#### 5.4.4. Ablation Study on the Roll Angle Smoothing Factor

To further evaluate the contribution of the roll angle smoothing factor, a partial ablation study was conducted under the same experimental setting. The complete ROIV-SLAM framework was compared with an ablated version, denoted as ROIV-SLAM w/o RSF, in which the roll angle smoothing factor was removed from the back-end factor graph while all other modules remained unchanged.

Both versions were evaluated using the same recorded sensor sequences to ensure a fair comparison. The experiments were conducted under both normal-disturbance and high-disturbance conditions. For each condition, three repeated trials were performed following the same protocol used in the previous experiments. The roll angle standard deviation, mean RSF residual, APE RMSE, and APE standard deviation were calculated to evaluate attitude stability, temporal smoothness, trajectory consistency, and repeatability, respectively.

The roll angle smoothing factor constrains the second-order temporal variation of the roll angle among three consecutive keyframes. Let $\alpha_{i-1}$, $\alpha_{i}$, and $\alpha_{i+1}$ denote the roll angles of three consecutive keyframes. The residual of the roll angle smoothing factor is defined as

(54) $$e_{\mathrm{RSF}}=\alpha_{i+1}-2\alpha_{i}+\alpha_{i-1}$$

The corresponding mean RSF residual is calculated as

(55) $${\bar{e}}_{\mathrm{RSF}}=\frac{1}{N-2}\sum_{i=2}^{N-1}\left|\alpha_{i+1}-2\alpha_{i}+\alpha_{i-1}\right|$$

where *N* denotes the number of keyframes. A smaller mean RSF residual indicates smoother roll angle variation and fewer non-physical attitude jumps.

The ablation results are summarized in Table 6 and Table 7, where “Roll Std.” denotes the roll angle standard deviation and “Mean RSF” denotes the mean roll angle smoot-hing residual.

As shown in Table 6, removing the roll angle smoothing factor leads to larger roll angle fluctuation and a higher mean RSF residual. This phenomenon is more obvious under the high-disturbance condition, where rapid roll angle variations are induced by external manual interference. The results indicate that the estimated roll angle becomes less temporally consistent when the smoothing factor is removed.

Compared with ROIV-SLAM w/o RSF, the complete ROIV-SLAM system achieves lower roll angle standard deviation and smaller mean RSF residual. This result indicates that the roll angle smoothing factor suppresses abrupt non-physical roll angle changes while preserving the overall motion trend of the robot. As shown in Table 7, the complete system also obtains lower APE RMSE and APE standard deviation, suggesting that improved roll angle stability contributes to better trajectory consistency with respect to the optimized reference trajectory.

**Figure 19:**
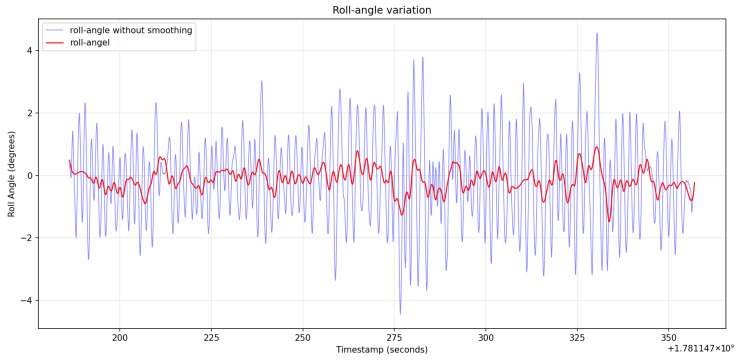
Roll angle variation with and without the roll angle smoothing factor under the high-disturbance condition.

Figure 19 shows the roll angle variation of ROIV-SLAM w/o RSF and the complete ROIV-SLAM system under the high-disturbance condition.

As shown in Figure 19, ROIV-SLAM w/o RSF exhibits more frequent abrupt fluctuations in the roll angle curve. In contrast, the complete ROIV-SLAM system produces a smoother roll angle trajectory while maintaining the real roll-motion trend caused by robot balancing. This suggests that the proposed smoothing factor does not over-constrain the robot motion, but mainly suppresses abnormal high-frequency attitude jumps.

**Figure 20:**
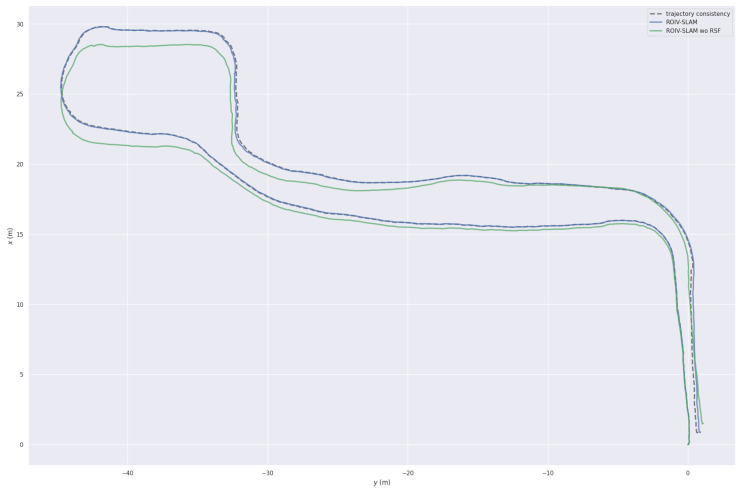
Trajectory comparison with and without the roll angle smoothing factor under the high-disturbance condition.

Figure 20 further compares the estimated trajectories of the two variants under the high-disturbance condition.

As shown in Figure 20, the trajectory estimated by ROIV-SLAM w/o RSF shows larger local deviations, especially in segments affected by strong roll disturbances. In contrast, the complete ROIV-SLAM system maintains better consistency with the optimized reference trajectory. These results indicate that the roll angle smoothing factor improves not only attitude stability but also trajectory consistency under high-frequency roll disturbances.

## 6. Discussion

The experimental results demonstrate that conventional single-modality SLAM systems and generic multi-sensor fusion frameworks encounter significant challenges when deployed on non-coaxial self-balancing robots operating under continuous high-frequency roll disturbances. Severe body oscillations may simultaneously degrade LiDAR, visual, and inertial observations. Point cloud distortion, IMU integration drift, and motion-induced image blur can collectively reduce the reliability of sensor measurements. Under such conditions, rotational errors may propagate into translational estimation through the coupled optimization process, resulting in trajectory drift and degraded mapping accuracy.

The proposed ROIV-SLAM framework addresses this challenge through a translation–rotation decoupled estimation strategy. Instead of jointly estimating all pose states in a conventional optimization framework, the system prioritizes rotation estimation on the manifold space by exploiting physically meaningful constraints derived from the gravity vector and RGB-D ground normal observations. By stabilizing attitude estimation before translational optimization, the proposed method effectively suppresses error leakage from rotation to translation and improves localization robustness under severe roll oscillations.

Furthermore, the roll angle smoothing factor incorporated into the back-end factor graph introduces temporal consistency constraints that are consistent with the continuous balancing behavior of the robot. Together with the vision–LiDAR dual-validation loop closure mechanism, the proposed framework achieves improved global consistency and reduces the influence of incorrect loop closures. Experimental results indicate that these design choices contribute to improved trajectory consistency and mapping robustness in curved corridors and highly disturbed environments. The ablation study further confirms the effectiveness of the roll angle smoothing factor. Compared with the ablated ROIV-SLAM variant without the roll angle smoothing factor, the complete ROIV-SLAM system achieves smoother roll angle evolution, lower mean RSF residuals, and improved trajectory consistency, especially under high-disturbance conditions. This indicates that the proposed smoothing factor does not merely serve as a heuristic filtering operation, but provides a physically meaningful temporal constraint that suppresses non-physical attitude jumps while preserving the continuous balancing motion of the robot.

Nevertheless, several limitations remain. Mechanical clearance variations and uneven contact conditions in curved corridors may further amplify roll disturbances and introduce additional uncertainty into sensor observations. In addition, reflective or transparent surfaces, such as glass curtain walls, may generate invalid LiDAR returns and unreliable depth measurements that cannot be completely removed by the current preprocessing pipeline. These factors may still cause local mapping inconsistencies and occasional pose estimation errors.

Another limitation of the current evaluation is the absence of direct comparisons with state-of-the-art 3D LiDAR–inertial and LiDAR–visual–inertial SLAM systems, such as LIO-SAM, FAST-LIO2, LVI-SAM, and R3LIVE. Although these methods have demonstrated excellent performance in large-scale mapping applications, they rely on sensing modalities that differ substantially from those available on the target platform. The proposed system is specifically designed for lightweight robotic platforms equipped with a 2D LiDAR, an RGB-D camera, an IMU, and wheel odometry, making direct experimental comparisons difficult without modifying the hardware configuration or introducing additional sensing assumptions.

A further limitation of the current evaluation is the absence of an independently measured ground-truth trajectory. The reference trajectory used in this study was generated through offline global optimization using the complete dataset collected during the experiment and therefore may contain residual estimation bias. Although this approach is commonly adopted in environments where external tracking systems are unavailable, it should be emphasized that the generated reference trajectory does not constitute absolute ground truth. Instead, it serves as a consistent benchmark for relative performance evaluation across different SLAM systems. Consequently, the reported Absolute Pose Error (APE) values should be interpreted as measures of trajectory consistency with respect to the optimized reference trajectory rather than absolute localization accuracy.

Although a partial ablation study has been added to evaluate the contribution of the roll angle smoothing factor, a more comprehensive module-level ablation analysis remains limited in the current study. The proposed ROIV-SLAM framework consists of several tightly coupled components, including EKF-based translation estimation, SO(3)-based rotation optimization, gravity- and ground-normal-based attitude constraints, the roll angle smoothing factor, and the vision–LiDAR dual-validation loop closure mechanism. In this revision, the roll angle smoothing factor was isolated and evaluated because it can be removed from the back-end factor graph while keeping the remaining system architecture unchanged. However, independently disabling other components, such as the gravity- and ground-normal-based attitude constraints or the decoupled translation–rotation estimation strategy, would require substantial modifications to both the front-end and back-end pipelines and may affect the stability of the entire system. Therefore, further systematic ablation studies are still required to quantify the relative contribution of each module in greater detail.

Future work will focus on more robust handling of reflective and transparent surfaces, adaptive confidence weighting for ground-normal estimation, and stronger motion priors derived from robot balancing controller states and platform dynamics. In addition, benchmark datasets, motion-capture systems, and alternative hardware configurations will be investigated to enable more rigorous quantitative evaluation and more comprehensive comparisons across heterogeneous sensing architectures. Although this study has provided a partial ablation analysis of the roll angle smoothing factor, more comprehensive ablation studies will be conducted in future work to further quantify the relative contribution of the remaining modules. These improvements are expected to further enhance the robustness, scalability, and generalization capability of the proposed system in complex real-world environments.

## 7. Conclusions

This paper proposes ROIV-SLAM, a rotation-optimized multi-sensor fusion SLAM framework designed for high-frequency roll disturbances encountered by non-coaxial two-wheeled robots. The proposed system adopts a translation–rotation decoupled front-end architecture. The translation component is estimated using an EKF framework that fuses LiDAR, IMU, and wheel odometry measurements, while the rotation component leverages gravity vectors and RGB-D ground normals to construct physical tangent-space constraints and performs optimization on the SO(3) manifold to support stable rotation estimation.

In the back-end, a sliding-window factor graph optimization framework is employed. A roll angle smoothing factor is introduced to suppress high-frequency roll estimation noise, and a visual–LiDAR dual verification loop closure mechanism is incorporated to improve global consistency. The added ablation study further indicates that the roll angle smoothing factor improves temporal roll angle consistency and reduces trajectory degradation under high-disturbance conditions.

Experimental results show that the proposed method achieves improved trajectory consistency with respect to the optimized reference trajectory compared with the evaluated baseline methods in the tested scenarios. The system also produces more coherent mapping results under high-frequency balancing motion, indicating improved robustness of state estimation for the target lightweight robotic platform.

Overall, the proposed approach provides a practical solution for robust SLAM in scenarios involving continuous roll disturbances and contributes to improving the trajectory consistency of self-balancing mobile robots under the tested sensing configuration.

Future work will focus on more comprehensive evaluations using benchmark datasets and independently measured ground-truth trajectories. In addition, more systematic module-level ablation studies will be conducted to quantify the relative contribution of the remaining components to overall system performance.

## Figures and Tables

**Table 1:** Trajectory Consistency Error and Algorithm Performance Comparison.

Metric	LiDAR SLAM	RGB-D SLAM	ROIV-SLAM
Mean APE (m)	7.21	4.01	0.15
Median APE (m)	8.29	4.68	0.16
APE RMSE (m)	8.48	4.57	0.19
APE Std. dev. (m)	4.45	2.18	0.11
Runtime (s)	221	248	236
Memory usage (MB)	420	630	675

**Table 2:** Average execution time of the main modules in ROIV-SLAM.

Module	Average Time (ms)
Front-end sensor processing	24.8
State estimation and roll angle optimization	11.6
Loop-closure detection	31.4
Back-end graph optimization	42.7

**Table 3:** Localization performance in different environments over repeated trials.

Environment	Method	APE RMSE (m)	Std. Dev. (m)
Looped corridor	Cartographer	5.91	3.59
Looped corridor	RTAB-Map	4.26	2.08
Looped corridor	ROIV-SLAM	0.27	0.18
Composite environment	Cartographer	13.44	7.20
Composite environment	RTAB-Map	3.69	2.31
Composite environment	ROIV-SLAM	0.97	0.58

**Table 4:** Roll angle variation under different disturbance conditions.

Disturbance Condition	Max. Roll Angle (deg)	Roll Angle Std. Dev. (deg)
Normal disturbance	2.34	1.04
High disturbance	9.88	3.64

**Table 5:** Localization performance under different roll-disturbance conditions.

Disturbance Condition	Method	APE RMSE (m)	Std. Dev. (m)
Normal disturbance	Cartographer	13.4	7.20
Normal disturbance	RTAB-Map	3.69	2.31
Normal disturbance	ROIV-SLAM	0.97	0.58
High disturbance	Cartographer	14.50	7.55
High disturbance	RTAB-Map	4.46	2.90
High disturbance	ROIV-SLAM	1.13	0.76

**Table 6:** Roll angle stability ablation results.

Condition	Method	Roll Std. (°)	Mean RSF (°)
Normal	ROIV-SLAM w/o RSF	1.36	0.17
Normal	ROIV-SLAM	0.36	0.013
High	ROIV-SLAM w/o RSF	3.11	0.37
High	ROIV-SLAM	0.65	0.02

**Table 7:** APE-based trajectory consistency ablation results.

Condition	Method	APE RMSE (m)	APE Std. Dev. (m)
Normal	ROIV-SLAM w/o RSF	2.72	1.22
Normal	ROIV-SLAM	1.21	0.57
High	ROIV-SLAM w/o RSF	3.87	2.04
High	ROIV-SLAM	1.53	0.81

## Data Availability

The data presented in this study are available from the corresponding author upon reasonable request.

## Abbreviations

The following abbreviations are used in this manuscript:

SLAM	Simultaneous Localization and Mapping
IMU	Inertial Measurement Unit
EKF	Extended Kalman Filter
RGB-D	Red Green Blue-Depth
LiDAR	Light Detection and Ranging
APE	Absolute Pose Error
RANSAC	Random Sample Consensus
PnP	Perspective-n-Point
BoW	Bag-of-Words
